# An experimentally informed statistical elasto-plastic mineralised collagen fibre model at the micrometre and nanometre lengthscale

**DOI:** 10.1038/s41598-021-93505-0

**Published:** 2021-07-30

**Authors:** Alexander Groetsch, Philippe K. Zysset, Peter Varga, Alexandra Pacureanu, Françoise Peyrin, Uwe Wolfram

**Affiliations:** 1 School of Engineering and Physical Sciences, Heriot-Watt University, Edinburgh, EH14 4AS UK; 2 ARTORG Centre for Biomedical Engineering Research, University of Bern, Bern, Switzerland; 3 AO Research Institute Davos, Davos, Switzerland; 4 European Synchrotron Radiation Facility, ID16A, Grenoble, France; 5 Université de Lyon, CNRS UMR 5220, Inserm U1206, INSA Lyon, UCBL Lyon 1, Creatis, Lyon, France

**Keywords:** Structural properties, Computational biophysics, Nanoscale biophysics, Bioinspired materials, Biomedical materials, Biomedical engineering, Bioinspired materials, Composites, Mechanical properties, Computational methods, Characterization and analytical techniques, Imaging techniques

## Abstract

Bone is an intriguingly complex material. It combines high strength, toughness and lightweight via an elaborate hierarchical structure. This structure results from a biologically driven self-assembly and self-organisation, and leads to different deformation mechanisms along the length scales. Characterising multiscale bone mechanics is fundamental to better understand these mechanisms including changes due to bone-related diseases. It also guides us in the design of new bio-inspired materials. A key-gap in understanding bone’s behaviour exists for its fundamental mechanical unit, the mineralised collagen fibre, a composite of organic collagen molecules and inorganic mineral nanocrystals. Here, we report an experimentally informed statistical elasto-plastic model to explain the fibre behaviour including the nanoscale interplay and load transfer with its main mechanical components. We utilise data from synchrotron nanoscale imaging, and combined micropillar compression and synchrotron X-ray scattering to develop the model. We see that a 10-15% micro- and nanomechanical heterogeneity in mechanical properties is essential to promote the ductile microscale behaviour preventing an abrupt overall failure even when individual fibrils have failed. We see that mineral particles take up 45% of strain compared to collagen molecules while interfibrillar shearing seems to enable the ductile post-yield behaviour. Our results suggest that a change in mineralisation and fibril-to-matrix interaction leads to different mechanical properties among mineralised tissues. Our model operates at crystalline-, molecular- and continuum-levels and sheds light on the micro- and nanoscale deformation of fibril-matrix reinforced composites.

## Introduction

Bone is an impressive, and complex hierarchical material made of abundantly available ingredients such as inorganic mineral nanocrystals and organic collagen molecules^1,2^. The hierarchical setup allows it to combine high toughness and strength in a lightweight design^3–6^, mechanical properties generally excluding themselves^7^. Relatively weak individual constituents are combined to produce outstanding apparent mechanical properties via a biologically driven self-organisation and self-assembly^2,8,9^. This inherently includes a certain degree of biological variability^10^ that can lead to increased nanoscale energy dissipation^11^. As a composite material, bone is multifunctional^3,12^, which also inspires the development and design of new metamaterials^2,9,12,13^. A detailed knowledge of bone is also fundamental to understand its underlying deformation mechanisms and, in turn, possible changes that arise due to bone-related pathologies such as osteoporosis, osteoarthritis, bone cancer or osteogenesis imperfecta (OI). Especially for diseases such as OI, where bone brittleness originates at the molecular level^14^, we need to study component-related changes (bone quality) and not only the bone quantity. It has been shown that by including information on the bone ultrastructure, we can also improve the outcome of computational models used in the diagnosis and risk prediction of osteoporotic patients^15–17^. Such models can be a cornerstone in tackling the socio-economic burden of bone diseases through improved diagnoses and custom implants^17–19^. However, these models critically depend on the underlying material description at lower length scales, and our knowledge is still limited, especially with respect to yield and post-yield properties beyond the elastic regime^20^.

At the micro- and nanoscale, the elementary mechanical units are the mineralised collagen fibre and mineralised collagen fibril^8,21^. A fibre is a fibril-matrix reinforced composite with an array of fibrils embedded in an extrafibrillar matrix (water, mineral, non-collagenous proteins (ncp))^1,8,22–24^. Fibrils combine type I collagen molecules and mineral particles of primarily carbonated hydroxyapatite^25,26^ with a staggered collagen arrangement^25–27^. The deformation and failure mechanisms of bone depend on the scale^1,8^. This includes an increasing strength and a brittle-to-ductile transition from macro- to microscale^28,29^ where the microscale ductility is attributed to the amount of extrafibrillar matrix shearing^10,30^. Understanding failure at the fibril- and fibril array-levels can help us to understand macroscopic yielding^31^. At the nanoscale, intrafibrillar deformation is reported to be a result of shear strain between mineral and collagen, shearing in the interparticle collagen matrix, stress transfer between adjacent particles and intrafibrillar sliding^32,33^. Extrafibrillar failure is predominantly related to shear deformation, friction of the extrafibrillar matrix^23,34^ and a failure at the fibril-matrix interface^35^, which eventually leads to macroscopic plastic strains^35^. Failure is also reported to be due to ductile sliding of mineral particles and rupture of collagen fibrils^36^ while micropillar compression of extracellular matrix bone samples showed zones of localised shear deformation^28,37^, which points to extrafibrillar sliding. Interfibrillar cross-links are further involved in forming sacrificial bonds which play a role in the toughening mechanisms of bone^23,38^. The predominant deformation and failure mechanisms are, thus, related to the shearing of the components and a failure at their interfaces.

**Figure 1:**
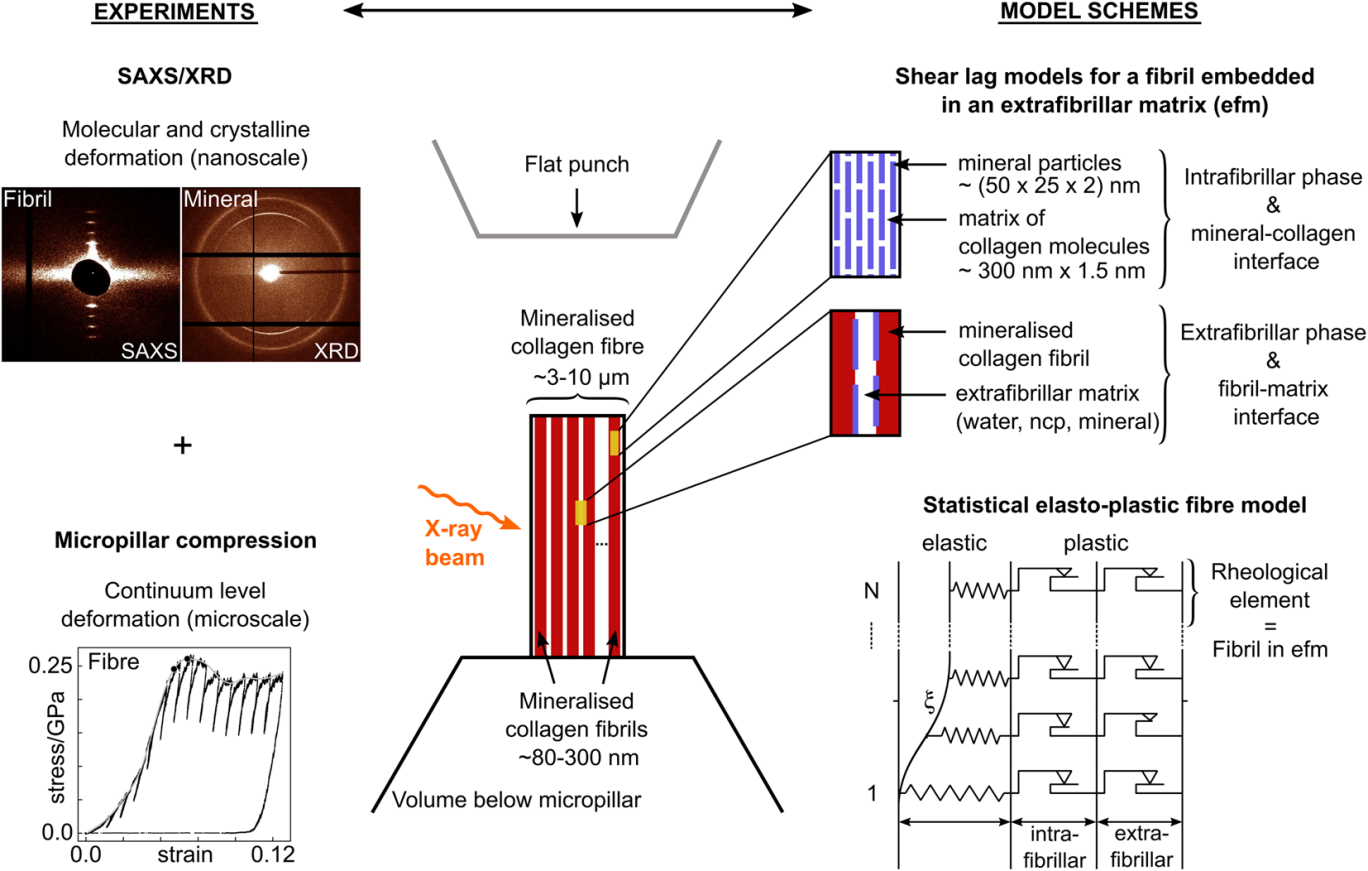
Development of the experimentally informed statistical elasto-plastic model for the mineralised collagen fibre at the micro- and nanoscale. The apparent fibre behaviour was quantified by compressing fibre micropillars (continuum level; microscale deformation) while determining molecular and crystalline deformations via fibril and mineral strains (nanoscale deformation) with small angle X-ray scattering (SAXS) and X-ray diffraction (XRD, WAXS)^39^. The elasto-plastic behaviour of intra- and extrafibrillar phases is modelled via two nested shear lag models and two inelastic strain mechanisms at mineral-collagen and fibril-matrix interfaces. The fibre itself is then represented by a parallel arrangement of fibrils with variable material properties where a rheological element represents a fibril embedded in an extrafibrillar matrix.

Experimentally, we recently presented micro- and nanomechanical data for individual mineralised collagen fibres^39^ (Fig. 1). We combined micropillar compression^28,40^ of 6 $\upmu \hbox {m}$ × 12 $\upmu \hbox {m}$ samples with synchrotron X-ray scattering/diffraction (SAXS/XRD)^27,31,32,41,42^ (beam size of 5.5 $\upmu \hbox {m}$ × 7.0 $\upmu \hbox {m}$) and quantified the apparent mechanical fibre behaviour as well as the mechanical interaction with fibrils and mineral particles. Until then, in situ mechanical and X-ray scattering experiments (e.g.^31,32,41,42^) only tested samples down to the millimetre length scale using larger beam sizes. In all these studies, overall strains for fibrils and mineral particles were smaller compared to the macroscopic tissue response. These small strains are usually explained by aforementioned dissipation mechanisms^23,31,32,34,39,43,44^. In our experiments^39^, testing single fibres allowed us to assume a parallel fibril arrangement while excluding the effect of interfaces above the fibre level. Consequently, we expected strains at the fibril- and fibre-level to be similar but we found large differences. Given the micrometre sized samples, this difference was even more severe as previously reported results (e.g.^31,32,41,42^). Although the experimental outcome contradicted our expectations, data from these length scales give us the unique possibility to identify the actual mechanical behaviour if this could be captured in a mathematical model.

Different analytical and computational models have been used to simulate bone tissue mechanics. Micromechanical homogenisation techniques are a convenient way to calculate the behaviour of mineralised tissues along multiple hierarchies but are restricted to the use of average stress values for different constitutive phases^15,30,36,45,46^. They cannot consider any biological heterogeneity and inter-phase stress transfer, partly resulting from the intrafibrillar staggered arrangement^26,33,47^. Cohesive finite element models increasingly aim to address the important interplay between the different phases of bone^48–50^. These models rely on a detailed multiscale material description, especially when it comes to the interfaces between constitutive phases. Molecular dynamics models^51^ can provide the important part that biochemistry plays in the material behaviour down to the atomistic scale. To truly mimic the biological tissue behaviour, we need to consider the heterogeneity in mechanical properties. One approach is to combine elementary rheological models^52,53^ with statistically distributed model parameters^28,54,55^. So far, these models explain the mechanics at the continuum level of bone extracellular matrix including an upscaling towards millimetre length scales^28,55^. But a model is missing that operates in between the molecular and continuum levels, which also incorporates the interaction between mechanical components. It should cover elasticity and plasticity, and allow us to compare deformation mechanisms and load transfer in different mineralised tissues. In composite materials such a load transfer can be simulated by shear lag models, which calculate the deformation between composite phases^26,47,56–59^. They have been successfully applied to bio-composites, especially to account for the intrafibrillar staggered collagen arrangement and the interaction of mineral particles within the protein matrix^26,58^. Yet no shear lag model for a mineralised collagen fibre exists that incorporates plasticity. It would enable us to study the elasto-plastic fibre behaviour and how its constituents contribute to its apparent behaviour. The statistical material description then covers the biological variability and opens the possibility to incorporate pathological changes in the simulation while generalising the model.

In this paper, we develop a 1D constitutive model for a mineralised collagen fibre that operates at the continuum-, molecular- and crystalline-levels covering fibre, fibrils, collagen molecules and mineral nanocrystals (Fig. 1). To simulate the intrafibrillar phase, we model the mechanics within a composite of collagen molecules and mineral particles. We embed this fibril into an extrafibrillar matrix, and simulate the elastic fibril behaviour within an organic matrix. To account for the predominant shear deformation within the fibre composite, we use shear lag models. To capture strength, we incorporate plasticity by using two inelastic strain mechanisms at the interfaces of mineral-collagen(-mineral) and fibril-matrix(-fibril) (Fig. 1). We then model the apparent fibre behaviour via a parallel arrangement of such fibrils with variable material properties. With this statistical material description, we study the effect of micro- and nanomechanical heterogeneity. We use synchrotron radiation phase-contrast nanometre computed tomography (SRnCT) to determine the composition of the model including tissue mineral density, fibril diameter and 3D fibril orientation. For the model development, we utilise our previous experimental data^39^, and show how we generalise it towards other fibril-matrix reinforced composites to understand and identify different micro- and nanoscale deformation mechanisms in mineralised tissues.

## Results

In the following we first present the modelling concepts including the use of two nested shear lag models for the intra- and extrafibrillar phases to calculate the elastic properties of a mineralised collagen fibril and fibre. We then incorporate plasticity by using an elasto-plastic rheological element that represents a mineralised collagen fibril embedded in an extrafibrillar matrix. A parallel arrangement of fibrils then represents the fibre mimicking the setup observed in Fig. 1. Material properties of these fibrils vary statistically. We further show how we use SRnCT to determine tissue mineral density, fibril diameter and local 3D fibril orientation. These quantities can be related to the material itself and were used in the model composition. We also quantified the surface roughness of the micropillars, a material independent structural parameter, as a result of sample fabrication. The surface roughness values allowed us to formulate a non-linear recruitment mechanisms of fibrils that influenced reported experimental results^39^.

### Statistical elasto-plastic fibre model at micro- and nanoscale

#### Two nested shear lag models for elasticity and load transfer

In our model, we want to cover the elasto-plastic behaviour of a fibre during compression. As outlined in the introduction, the predominant micro- and nanoscale deformation and failure mechanism of bone is shearing between the constitutive phases^23,28,32–37^. Within a mineralised collagen fibril, we focus on the interface between the collagen molecules and mineral particles (Fig. 1, intrafibrillar phase), outside the fibril on the interface of fibril and extrafibrillar matrix (Fig. 1, extrafibrillar phase). To calculate the elastic properties in these two phases, we make use of the concept of shear lag models^26,31,47,56,57,59–61^. The basic assumption of these models is that an externally applied load is transferred between the constitutive phases through shear stresses while we deal with a stiff and a compliant part within a composite. This can be related to the elastic modulus of the stiffer phase being at least one order of magnitude larger than the shear modulus of the compliant phase. In our case, we use two shear lag models, one for the intrafibrillar phase where the stiff mineral particles are embedded in an organic matrix of collagen molecules, and one for the extrafibrillar phase where the stiffer mineralised collagen fibril is embedded in an extrafibrillar matrix. Stress between the load bearing stiffer parts can only be transferred via shear through the compliant matrix. Under these assumptions, we calculate the elastic modulus of a mineralised collagen fibril and a fibril embedded in the matrix (fibre) (Eqs. (4) and (6) in “Methods”). Based on these values, we can calculate the mineral and fibril strains (Eq. (5) in “Methods”). Details on the mathematical formulation and derivation of the equations for the shear lags and an overview of the model parameters are provided in the Methods sections "Two nested shear lag models for a mineralised collagen fibre" and “Model parameters” including Table 2. The composition of the model, i.e. the mineral volume fraction and the fibril volume fraction, is based on SRnCT measurements (Results section “Synchrotron radiation phase-contrast nanometre tomography” and Methods section “SRnCT of the ultrastructure of a mineralised collagen fibre”).

#### Elasto-plastic rheological unit and parallel arrangement with statistical description

**Figure 2:**
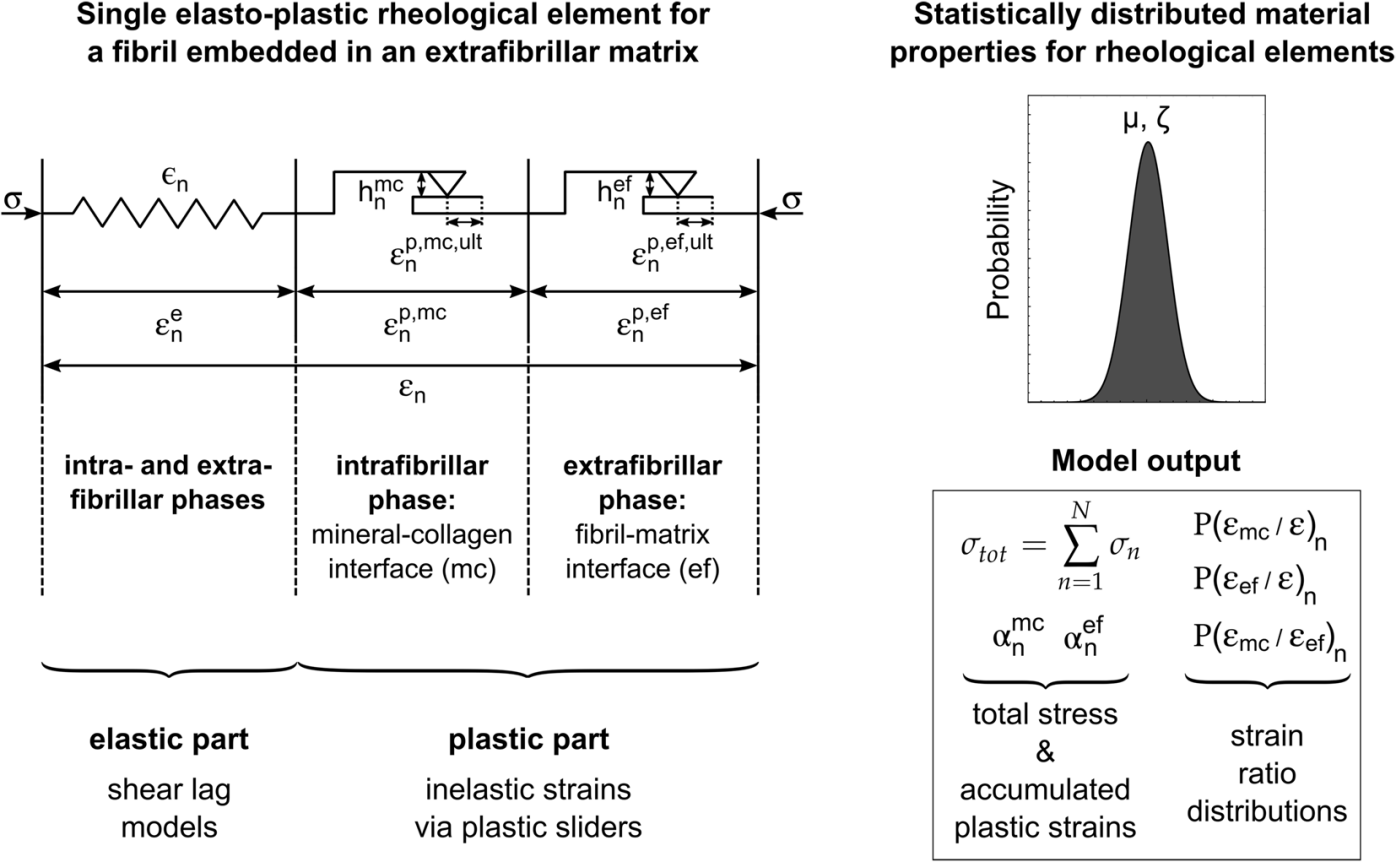
Elasto-plastic rheological model element to incorporate two inelastic strain mechanisms for the mineral-collagen (intrafibrillar) and fibril-matrix (extrafibrillar) interface coupled with a statistical material description. Left, a single rheological model element combines an elastic spring and two plastic sliders for these interfaces, which are set in series, and are loaded under a compressive stress $\sigma$. The series arrangement reflects the underlying shear lag and allows different strains in the intra- and extrafibrillar phases. $\epsilon$ denotes the apparent fibre Young’s modulus, which is calculated via the shear lag models. Linear hardening is considered via $h^{mc}$ and $h^{ef}$. The plastic sliders are deactivated when they reach ultimate strain values $\varepsilon ^{p,mc,ult}$ or $\varepsilon ^{p,ef,ult}$, respectively A parallel arrangement represents a fibre (Results section "Statistical material properties" and Fig. 1). Right, the mechanical properties of single elements varied statistically to simulate the effect of micro- and nanoscale heterogeneity (Methods sections “Elasto-plastic constitutive model for a fibril array”  and "Model parameters" including Table 2). The model outputs the total stress as the sum of the partial stresses (Supplementary Sect. S1), accumulated plastic strains in the intra- and extrafibrillar phases, and strain ratio distributions between the apparent fibre and its constitutive phases.

To extend the shear lag models towards plasticity and to calculate strength, we introduce an elasto-plastic rheological unit that represents a mineralised collagen fibril embedded in an extrafibrillar matrix (Figs. 1 and 2). A single rheological unit (Fig. 2, left) consists of an elastic spring, representing the apparent modulus of the intra- and extrafibrillar phases (Eq. (6) in “Methods”), set in series with two plastic sliders, one for the intrafibrillar interface of mineral-collagen-mineral and one for the extrafibrillar interface of fibril-matrix-fibril (Eqs. (10) and (11) in "Methods"). We can calculate accumulated plastic strains in the intra- and extrafibrillar phases (Eqs. (17) and (18) in "Methods") while the plastic sliders for these phases break down when reaching their ultimate value ($\varepsilon ^{p,mc,ult}$ and $\varepsilon ^{p,ef,ult}$ in Fig. 2 and Table 2 in “Methods”). When this happens, the rheological element is deactivated (Eqs. (25) and (26) in “Methods”), which means that the fibril fails. The series arrangement of the intra- and extrafibrillar phase reflects the underlying shear lag model with a stiff fibril in series with a shearing matrix. And it is motivated by the different amount of strain observed in the two phases when applying an apparent load at a higher hierarchical level^31,33,39,41^. The decomposition of the fibril strain into an elastic and plastic part^62^ allows us to calculate a ratio between the totally applied fibre strain, apparent fibril strain and mineral particle strain (Eqs. (8) and (9) in “Methods”). With this approach we incorporate two inelastic strain mechanisms while considering the main deformation via shearing in the constitutive phases (shear lag models). The mathematical formulation with a detailed derivation of the governing equations is given in "Methods" (section "Analytical mathematical framework for a single rheological element"). The numerical algorithm can be found in the Supplementary Sect. S1.

To represent a mineralised collagen fibre, we use a parallel arrangement of such rheological elements (Figs. 1 and 2) to model a parallel fibril array. We use SRnCT to confirm this arrangement (Results section "Parallel fibril arrangement within a fibre" and Methods section "Local 3D fibril orientation"). With SRnCT, we also determine the fibril diameter and, in turn, the number of fibrils (618 rheological elements) within a fibre (Results section "Fibril diameter and number of rheological elements" and Methods section "Fibril diameter, packing density and number of rheological units"). Since the model elements are parallelly arranged, the total stress is the sum of the individual stresses $\sigma _{i+1}^{tot} = \sum _{n=1}^{N} \sigma _{i+1,n}$ of N fibrils embedded in an extrafibrillar matrix (Supplementary Sect. S1). We, thus, can vary their material properties to study the effect of micro- and nanoscale heterogeneity. This also means that single model elements can have zero stress (deactivated fibrils). We incorporate this heterogeneity by using statistical distributions for mechanical model parameters (Methods sections "Statistical material properties" and "Model parameters" including Table 2). The model outputs the total stress $\sigma _{tot}$, the accumulated plastic strains in the intra- and extrafibrillar phases $\alpha ^{mc}$ and $\alpha ^{ef}$ as well as the statistical distributions for the strain ratios between mineral particles, mineralised collagen fibrils and the apparent fibre response ($P(\varepsilon _{mc}/\varepsilon )_n$, $P(\varepsilon _{ef}/\varepsilon )_n$ and $P(\varepsilon _{mc}/\varepsilon _{ef})_n$) (Fig. 2). Effectively, we can simulate a fibril-matrix-reinforced composite which can be applied to similarly structured materials (see "Discussion and conclusion"). Since fibril and mineral strains from in situ micropillar compression and SAXS/XRD^39^ represent average values within the illuminated X-ray beam volume, we can directly relate the model outcome to such experiments. Yield and ultimate values for the model are based on experiments^39^ and the literature^33,63^. A full list of model parameters is given in Table 2 of the Methods section “Model parameters”.

### Synchrotron radiation phase-contrast nanometre tomography

To determine tissue mineral density, fibril diameter and local 3D fibril orientation, we used synchrotron radiation phase-contrast nanometre computed tomography (SRnCT) (isotropic voxel size (20 nm)^3^) for six of the compressed fibre micropillars^39^. With SRnCT, we can detect small structural changes and, when using a monochromatic X-ray beam, an absolute value for the local tissue mineral density at the micro- and sub-microscale^64–68^ (Methods section “SRnCT of the ultrastructure of a mineralised collagen fibre”). These three quantities are related to the material itself and were used to define the fibre composition (Methods section "Two nested shear lag models for a mineralised collagen fibre" and Table 2). Together with the fibre diameter^39^, the information on the fibril diameter was used to determine the number of rheological model elements. The fibril orientation allowed us to confirm the parralel arrangement of fibrils and model elements. The sample surface roughness was assessed as a material independent parameter, which was a result of the micropillar fabrication process^39^. This surface roughness had an effect on the apparent stress-strain data and the fibril and mineral strains as seen in experiments^39^. To account for this artefact in the model, the surface roughness was used to identify a gradual and non-linear recruitment function for mineralised collagen fibrils (Results section "Heterogeneous deformation of fibrils due to their gradual recruitment" and Methods section "Surface roughness and fibril recruitment").

**Figure 3:**
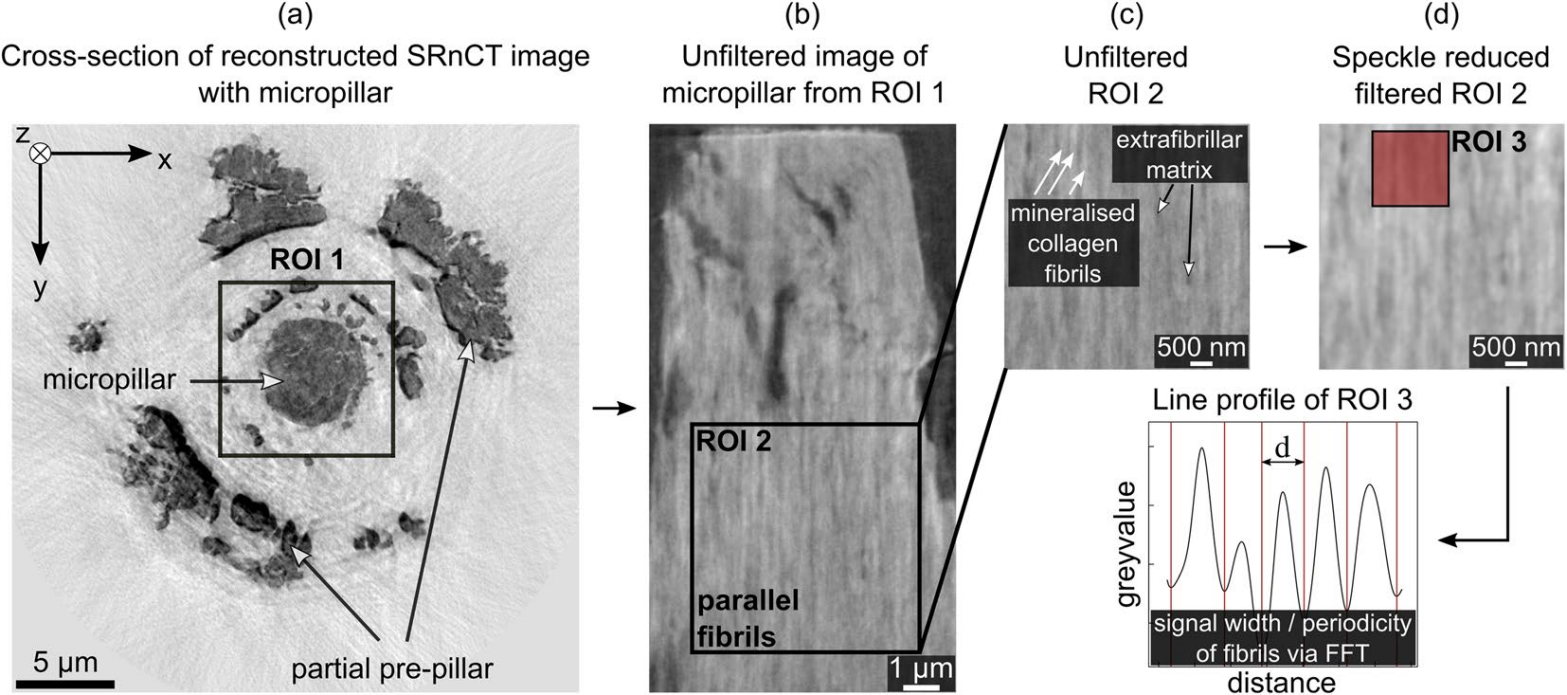
Extraction of the mineralised collagen fibril diameter based on reconstructed SRnCT images. (**a**) A region of interest (ROI 1) around the micropillar (post test) from cross-sections was chosen for further image processing and analyses. (**b,c**) Brighter regions in ROI 2 of the inverted image are related to mineralised collagen fibrils, darker regions to extrafibrillar matrix. (**d**) An adaptive edge-sensitive speckle reducing anisotropic diffusion^69^ was used for image enhancement in Fiji (imageJ v1.53c)^70^. The signal width *d* for 81 mineralised collagen fibrils and the periodicity for 23 line profiles in ROI 2 related to the mineralised collagen fibrils were analysed via local minima and Fast Fourier Transform (FFT), respectively, implemented in R (V3.6.2).

#### Tissue mineral density within a fibre

Especially for the elastic stiffness of mineralised tissues, the degree of mineralisation plays an important role^15,26,71^. We determined the tissue mineral density $\rho$ in $\hbox {g/cm}^{3}$ within the micropillars to inform the composition of the shear lag models (Methods section "Two nested shear lag models for a mineralised collagen fibre"). We also use the results to generalise the model while comparing them to values of other fibril-matrix reinforced composites (see “Discussion and conclusion”). We determined the local mass density of each voxel within cubic sub-volumes of the micropillar based on the analysis of grey value distributions in the reconstructed SRnCT images and used Guinier's approximation^67,72^. Detailed information is provided in the “Methods” section and Supplementary Sect. S3 with Figs. S7 and S8.

We found a mean tissue mineral density (mineralisation) within the micropillar area of $1.64 \pm 0.16\, \hbox {g/cm}^{3}$ ($\hbox {N}=6$). Minimum, median and maximum values were $1.41 \,\hbox {g/cm}^{3}$, $1.65 \,\hbox {g/cm}^{3}$ and $1.86 \,\hbox {g/cm}^{3}$, respectively.

#### Fibril diameter and number of rheological elements

To determine the number of parallelly arranged fibrils within a fibre (Fig. 1), we analysed line profiles from sagittal views of reconstructed SRnCT images (Fig. 3b) in regions of interest (5.5 $\upmu \hbox {m}$ × 5.5 $\upmu \hbox {m}$) within the undeformed part of the micropillar. Brighter regions in the inverted image are related to the mineralised collagen fibrils, the darker regions to the extrafibrillar matrix. Signal width and periodicity related to the mineralised collagen fibrils were then extracted based on local minima and by calculating the Fast Fourier Transform (FFT), respectively, using Fiji (imageJ v1.53c)^70^ and R (V3.6.2)^73^. A total of 81 fibrils were analysed.

The FFT line profile analysis returned a mean fibril diameter of $218 \pm 30$ nm, the analysis of the local minima $230 \pm 34$ nm, yielding an combined value of $224 \pm 45$ nm. This value was used in further calculations. Based on the average micropillar diameter in the experiments of $6.08 \pm 0.83$ $\upmu \hbox {m}$, a closed packing density of 0.86 was calculated. This led to $618 \pm 213$ fibrils within the micropillar. Thus, 618 single rheological model elements were used during the simulation. Details can be found in the Methods section "Fibril diameter, packing density and number of rheological units".

#### Parallel fibril arrangement within a fibre

To justify the parallel fibril arrangement in our model and to work with an explicit arrangement of rheological model elements, we quantified the fibril 3D orientation using an auto-correlation based measure^74^ (Methods section "Local 3D fibril orientation"). Cubic sub-volumes ($60 \,\hbox {voxels} = (1.2\,\upmu \hbox {m})^{3}$) within the undeformed part of the micropillar and volume below were used to determine the fibril orientation of two previously compressed micropillars. SEM images and the parallel arrangement visible in SRnCT were comparable for all other specimen (SEM: $\hbox {N}=11$; SRnCT: $\hbox {N}=6$). The calculation of the off-axis angle of the fibril vector orientations relative to the longitudinal fibre direction gave a mean value of $1.93 \pm 4.49^{\circ }$ in the undeformed part of the micropillars and $1.98 \pm 3.31^{\circ }$ in the volume below. The longitudinal structure confirms the uniaxial arrangement within the micropillars^39^ with a local 3D measurement and justifies using a parallel fibril arrangement in the model. A 3D representation of the fibril orientation is provided in Supplementary Sect. S3.2 including Fig. S9.

### Micro- and nanomechanical experiments

To compare the simulated mineralised collagen fibre to experiments we use our previously reported results from in situ micropillar compression and SAXS/XRD^39^. Briefly, we tested micropillars with a height of 12 $\upmu \hbox {m}$ and a diameter of 6 $\upmu \hbox {m}$ that we extracted from individual mineralised collagen fibres. We used mineralised turkey leg tendon, widely used as a model system for bone due to its uniaxial fibre arrangement and same structural set-up at the fibre level^15,22,27,75–77^. For sample preparation, the uniaxial fibre arrangement was essential to centre the preparation on individual fibres. Details on the sample preparation can be found in Ref.^39^ and in the Methods section "Extraction of mineralised collagen fibre micropillars". The micropillars were compressed using a cyclic quasi-static loading protocol up to 12% of strain including failure while fibril and mineral strains were assessed at 120 discrete time points via SAXS/XRD. Stiffness, yield point, and strength were extracted at the apparent fibre level (microscale). The mechanical interaction with the fibre nanoscale components, i.e. mineralised collagen fibrils and mineral particles, was quantified by calculating strain ratios between fibril-to-fibre, mineral-to-fibre and mineral-to-fibril that explain how components deform under a given microscopic load. Ratios of strain of 22:5:2 were measured between the fibre-, fibril- and mineral-levels. The model response was verified against these micro- and nanomechanical test results^39^. They included the fibre Young’s modulus of $16.47 \pm 3.40\,\hbox {GPa}$ (last unloading segment before yield), a fibre yield stress of $0.154 \pm 0.051\,\hbox {GPa}$, a compressive strength of $0.180 \pm 0.042\,\hbox {GPa}$ and the plastic strain of 0.08 where damage occurred the first time. At the apparent yield point of 0.04 we found a strain ratio for fibril-to-fibre of $0.20 \pm 0.17,$ and mineral-to-fibre of $0.09 \pm 0.02.$

### Simulated mineralised collagen fibre and comparison with experiments

We quantified the surface roughness of the micropillars as a material independent parameter from the SRnCT dataset (Results section “Synchrotron radiation phase-contrast nanometre tomography”). This was necessary to account for an experimental artefact as a result of it. Using the surface roughness we defined a non-linear function for the gradual fibril recruitment (Results section “Heterogeneous deformation of fibrils due to their gradual recruitment” and Methods section “Surface roughness and fibril recruitment”). To illustrate the model outcome when we disregard or consider this fibril recruitment, we present both. Accounting for the fibril recruitment, we achieve a very good agreement with experiments (Table 1), which allows us to consider the model as verified. This, in turn, gives us the possibility to deduce the actual non-linear fibre behaviour by excluding this structural artefact. A detailed comparison of both model versions at specific loading steps can be found in Supplementary Sect. S2 with full loading cycles in Supplementary Videos 1 and 2.

**Figure 4:**
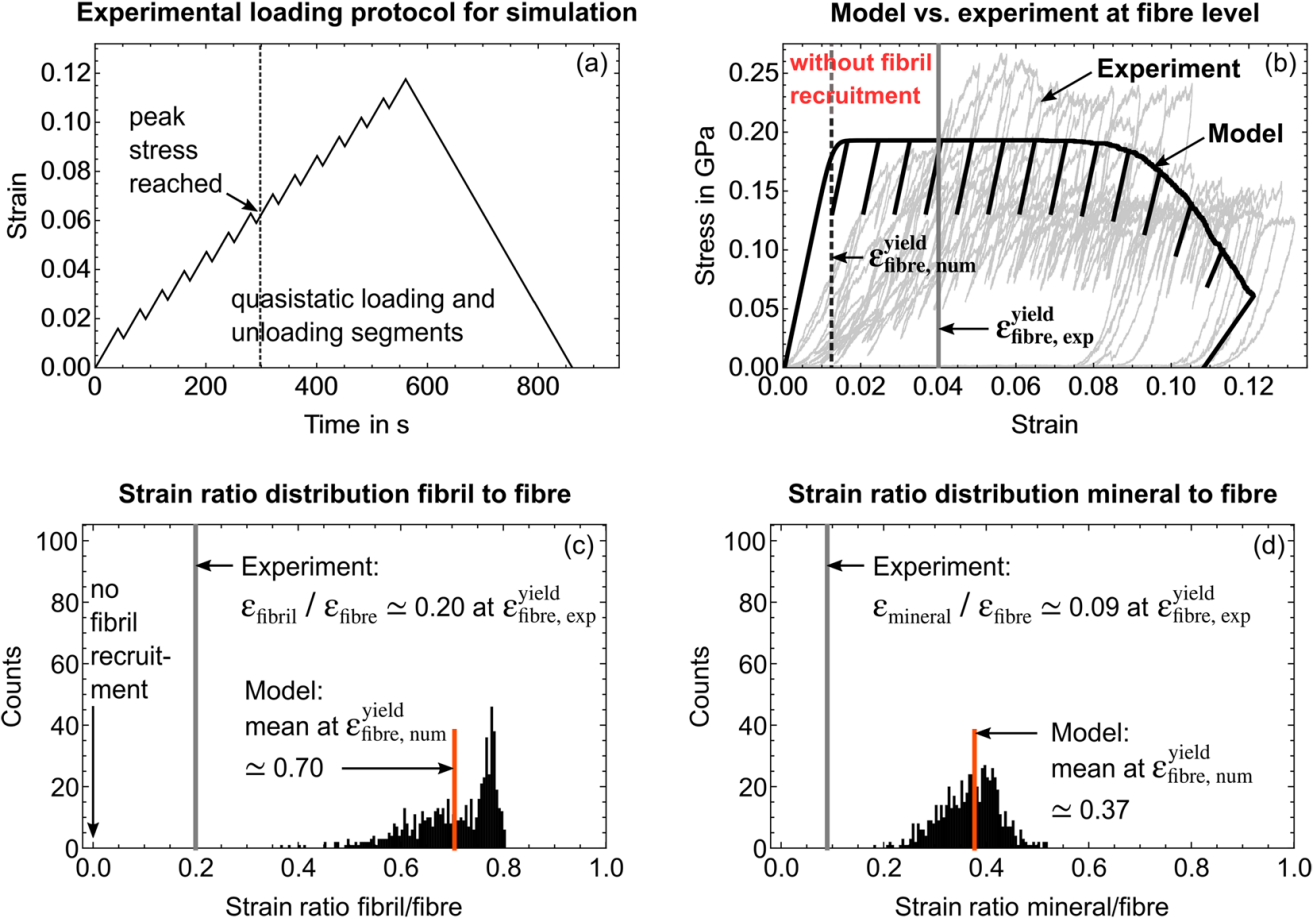
Model outcome for a simulated mineralised collagen fibre where we disregard non-linear fibril recruitment. Grey curves and lines represent experimental values^39^. (**a**) Experimental loading protocol. (**b**) Overlay of simulated stress-strain curve (black) and 11 experimental curves (grey) from fibre compression^39^. (**c,d**) Simulated strain ratio distributions for (**c**) fibril-to-fibre and (**d**) mineral-to-fibre at the simulated yield strain of 1.3% (black dotted line). The full loading cycle is presented in Supplementary Video 1.

#### Simulated fibre without fibril recruitment

Using the shear lag models we calculated an apparent fibre Young’s modulus of 15.803 GPa. The elasto-plastic model without fibril recruitment then simulated a mineralised collagen fibre yield stress of 0.184 GPa, a yield strain of 0.013 and a compressive strength of 0.189 GPa (Fig. 4). Fibril failure was seen from $0.06\,\upmu \hbox {m}/\upmu \hbox {m}$ accumulated plastic strain onwards. At the numerical yield point, the fibril-to-fibre strain ratio was 0.702, the mineral-to-fibre strain ratio 0.369. Results showed an initial fibril-to-fibre strain ratio of 0.8 which tends towards 1.0 and which would be expected in a parallel arrangement of rheological elements (Supplementary Video 1). The model agrees with experiments regarding the fibre Young’s modulus and compressive strength but not with respect to yield values and strain ratios. The apparent fibre failure also occurs earlier than in experiments (Fig. 4 and Supplementary Video 1).

**Figure 5:**
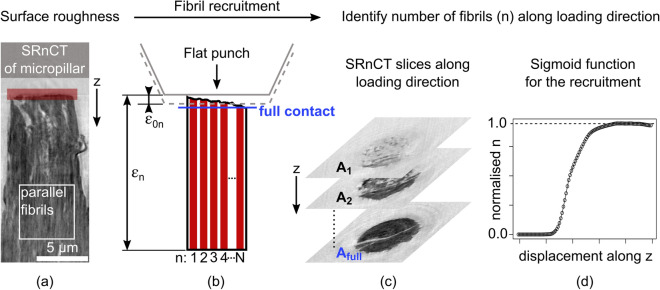
Quantification of the micropillar surface roughness to identify a non-linear function for the gradual fibril recruitment. (**a**) Reconstructed SRnCT image of a compressed micropillar with focus on the top part (red). Axial slicing and loading had the same direction denoted by z. (**b**) A diamond flat punch compresses a micropillar with N fibrils. The total strain $\varepsilon _n$ that is experienced by fibril *n* depends on its relative position to the flat punch. The corresponding fibril-specific strain offset value $\varepsilon _{0n}$ is then used in the $\varepsilon _n$ calculation. (c) The grey-value areas ($\hbox {A}_{1}$, $\hbox {A}_{2}$... $\hbox {A}_{{\text{full}}}$) related to the micropillar in the cross-sectional SRnCT slices were used to calculate area fractions relative to the point of full contact between flat punch and micropillar. (**d**) Based on the fibril volume fraction, a normalised number of fibrils along the loading direction was calculated and fitted with a non-linear function in R (V3.6.2)^73^.

#### Heterogeneous deformation of fibrils due to their gradual recruitment

To account for the experimental artefact of a gradual fibril recruitment during micropillar compression as a result of surface roughness, SRnCT reconstructions (Fig. 5a) were analysed. During such an experiment (Figs. 1 and 5), a flat diamond punch compresses the micropillar, in our case from a single mineralised collagen fibre (Figs. 1 and 5b). The location of the punch and the micropillar height are used to calculate apparent fibre strain $\varepsilon$. Due to the surface roughness fibrils were recruited gradually as the flat punch moved downwards. The total strain experienced by each fibril (rheological element *n* in the model), then depends on its relative position to the flat punch (Fig. 5b). To include this mechanism in the model, we needed to correct the total strains by an element-specific offset value $\varepsilon _{0n}$. Since this offset depends on the position of the flat punch relative to the micropillar surface, SRnCT images corresponding to the points from first (A_1_) to full contact (A_full_) were used. Grey-value areas from mineralised tissue in the cross-sectional SRnCT slices (Fig. 5c) were extracted by image thresholding, and edge detection using an active contour model, implemented in Python (v2.7)^78^ and Fiji (imageJ v1.53c)^70^. These areas were related to the number of fibrils during the loading steps via voxel size and fibril diameter. We found that this non-linear recruitment follows a sigmoid function $\xi (\varepsilon )$ (Fig. 5d, Eq. (27)  and Figs. 1, 9a,b) and that only 20 out of 618 fibrils were recruited instantaneously when the fibre was compressed (Fig. 9c,d). More details and mathematical formulations are provided in the Methods section “SRnCT of the ultrastructure of a mineralised collagen fibre”. Supplementary Video 3 also provides a cross-sectional slicing through a compressed micropillars.

#### Simulated fibre with fibril recruitment

**Figure 6:**
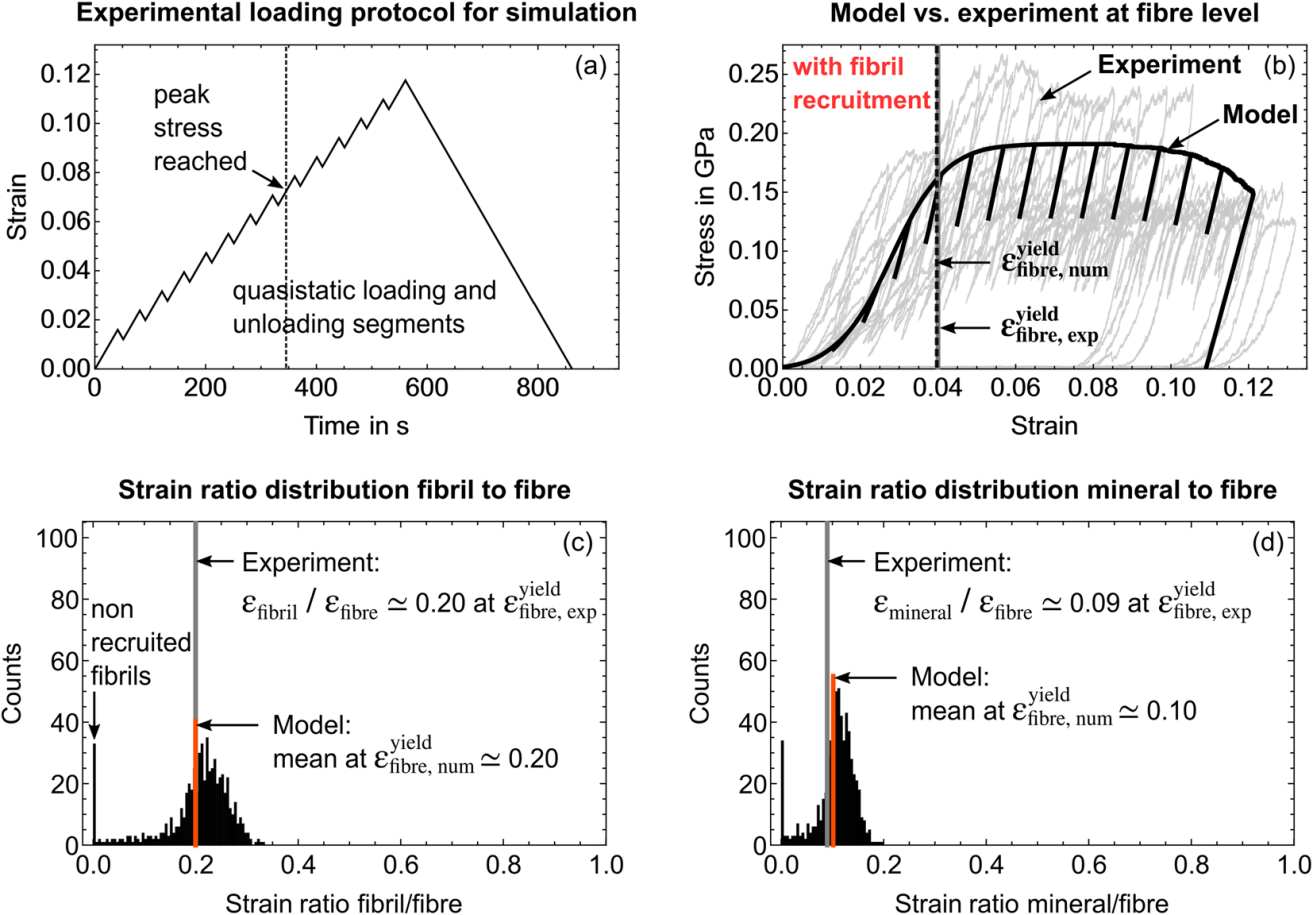
Model outcome for a simulated mineralised collagen fibre where we consider non-linear fibril recruitment. Grey curves and lines represent experimental values^39^. (**a**) Experimental loading protocol. (**b**) Overlay of simulated stress-strain curve (black) and 11 experimental curves (grey) from fibre compression^39^. (**c,d**) Simulated strain ratio distributions for (**c**) fibril-to-fibre and (**d**) mineral-to-fibre at simulated yield strain of 3.9% (black dotted line) with still 35 non recruited fibrils (**c**). A comparison between model and experiment is given in Table 1. The full loading cycle is provided in Supplementary Video 2.

The following model outcome considers the gradual and non-linear fibril recruitment. The only difference to the model where we disregard the recruitment is that the surface roughness was set to zero in the previous non-recruitment calculations. All other parameters stayed the same (see Table 2 in Methods section "Model parameters"). We will see that the surface roughness leads to a heterogeneous deformation and especially affects the initial region of the apparent stress-strain data as well as the strain ratios between the constitutive phases at the numerical yield point (Fig. 6). Since we use the same composition (material dependent parameters), the shear lag models computed an apparent Young’s modulus of the fibre of 15.803 GPa. The elasto-plastic model then simulated a mineralised collagen fibre with a yield stress of 0.162 GPa, a yield strain of 0.040 and a compressive strength of 0.188 GPa. Fibrils failed from $0.075~\upmu \hbox {m}/\upmu \hbox {m}$ accumulated plastic strain onwards. At the yield point, the mean for the fibril-to-fibre strain ratio was 0.199 and for the mineral-to-fibre strain ratio 0.100. A broad distribution of the strain ratios were found already in the initial phase confirming a very heterogeneous loading of the fibrils within the fibre (Supplementary Sect. S2 and Video 2). The distribution narrows with an increased compressive load and an increased number of recruited fibrils. This corresponds to a more distinct maximum of the histogram, especially after all fibrils have been recruited. This also corresponds to the transition from an initially heterogeneous deformation towards a more homogeneous strain ratio distribution. We observe this homogeneous deformation especially between the yield point and the point of compressive strength. The first fibrils fail shortly after full recruitment has been reached (Supplementary Video 2). We also see that the mineral particles strain is around 45% of the overall fibril strain. We reach a very good agreement with experiments at the micro- and nanoscale^39^ with an average of $94.9 \pm 3.8$% (Table 1 and Results section “Micro- and nanomechanical experiments”).

**Table 1:** Comparison of experimental^39^ ($\hbox {N=}11$) and simulated values from the statistical constitutive model for micro- and nanomechanical fibre properties when considering fibril recruitment.

	$\epsilon _{fibre}$/GPa	$\sigma _{33}^{yield}$/GPa	$\sigma _{33}^{str}$/GPa	$\varepsilon _{33}^{fibril}/\varepsilon _{33}^{fibre}$	$\varepsilon _{33}^{mineral}/\varepsilon _{33}^{fibre}$
Experiment	16.470	0.154	0.180	0.200	0.090
Model	15.803	0.162	0.188	0.199	0.100
Agreement	96.0%	94.8%	95.6%	99.5%	88.9%

#### Micro- and nanomechanical heterogeneity promotes microscale ductility

**Figure 7:**
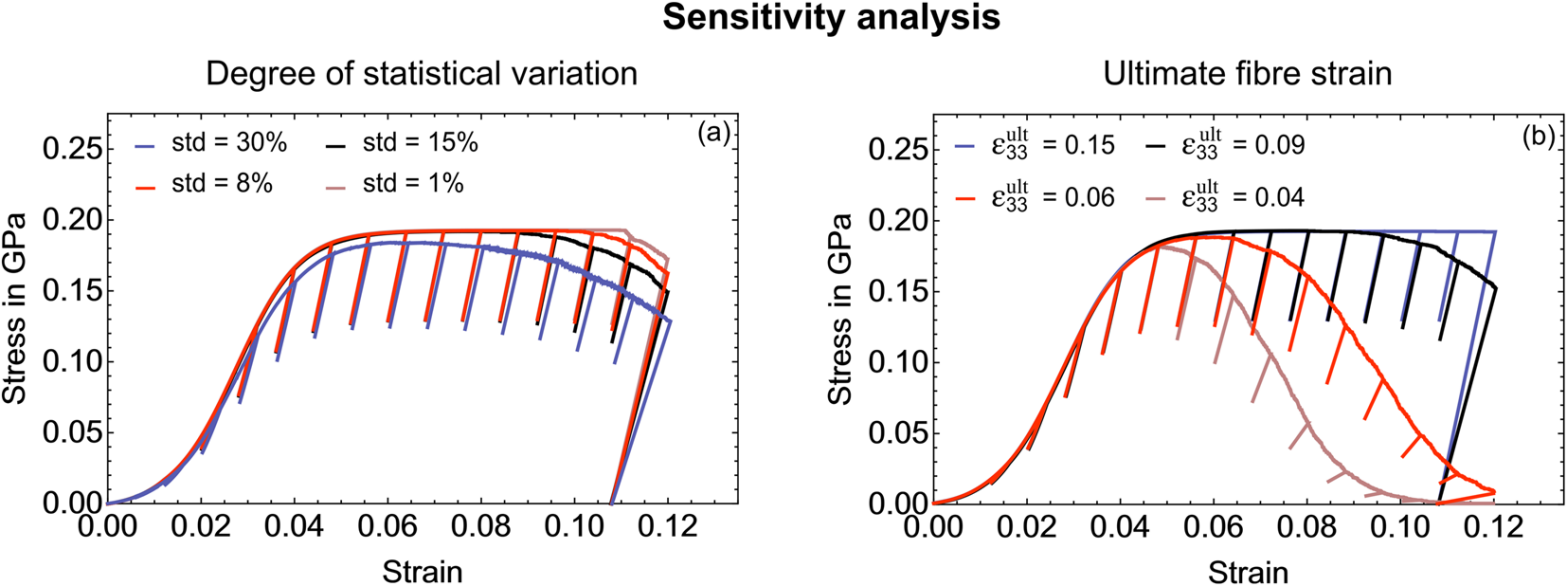
Sensitivity analysis of the elasto-plastic fibre model. (**a**) Influence of the amount of statistical variation of material properties (micro- and nanomechanical heterogeneity): A standard deviation of 15% for the normal distributions was used as a base value (see Methods section "Statistical material properties") and varied between 1 and 30%. (**b**) Influence of ultimate strain values (base value 0.09) (see Methods section "Model parameters" including Table 2).

We used our model to study the influence of the amount of micro- and nanomechanical heterogeneity on the overall fibre behaviour. For this, we varied the standard deviation of the model input values for elasticity (shear lag models) and plasticity (elasto-plastic rheological elements). Values included the base values of 15%, 8%^28,55^ plus a very small and very large value (1% and 30%) (see "Methods") . A smaller value corresponds to a smaller heterogeneity. At the lower end, i.e. at 1%, the model calculated a fibre with higher accumulated plastic strains and a more immediate overall failure (Fig. 7a) contrary to experiments^39^, preventing a prolonged ductile behaviour. At 30% standard deviation, we simulate a fibre in which the first fibrils fail earlier (ultimate strain shifts from 9% to 7%) next to increased softening (Fig. 7a). As in experiments^39^, unloading moduli stay the same pointing to an absence of damage. In contrast, changing the ultimate strain directly (Fig. 7b) leads to a more abrupt failure, a distinct softening and decreasing unloading moduli after first model elements fail, which disagrees with experiments^39^.

To confirm that using a normal distribution as statistical input represents the microscale heterogeneity in our model best (see Methods section "Statistical material properties"), we quantified the effect that skewed distributions have on the simulated fibre behaviour (Supplementary Sect. S4). Based on all mechanical data including our own experiments, and the used parameters in our model, we assume a normal distribution. The variation analysis shows that skewed distributions do not result in significantly different strain ratios or stiffness and only minor changes in strength. We conclude that skewed distributions do not present an advantageous set of properties for the material system (Supplementary Fig. S10).

## Discussion and conclusion

The aim of the study was to develop a model that captures the compressive elasto-plastic behaviour of a mineralised collagen fibre, bone’s fundamental mechanical unit. We used two nested shear lag models for the intra- and extrafibrillar phases to calculate elasticity at fibril- and fibre-level. We coupled these shear lag models with a parallel arrangement of elasto-plastic rheological elements to include two inelastic strain mechanisms at the mineral-collagen and fibril-matrix interfaces, and to represent the fibril arrangement within a fibre. The mechanical properties of model elements varied statistically. We utilised data from micro- and nanomechanical experiments, and nanoscale imaging to inform the model. It correctly simulates the fibre behaviour at the micro- and nanometre length scales as seen in experiments^39^ (Figs. 4 and 6). Thus, it captures the mechanical interplay between its constitutive phases bridging the crystalline, molecular and continuum levels. The statistical description allowed us to study the influence of micro- and nanomechanical heterogeneity on deformation and failure mechanisms at these length scales.

Using statistically varying mechanical properties for individual model elements (Figs. 1 and 2) gives us the flexibility to explore what happens to the overall system when individual units fail. Mathematically, this is possible due to the parallel arrangement where the total stress is the sum of the individual element stresses covering intra- and extrafibrillar phases. Since a single element represents a fibril embedded in an extrafibrillar matrix, during compression individual fibrils within the fibre can fail earlier than others corresponding to zero stress. This poses an advantage to continuum micromechanics^15,30,36,46^ where the behaviour in different constitutive phases are based on average stress values therein. By using shear lag models^26,33,47,59^ we account for the main micro- and nanoscale deformation mechanisms of bone^23,28,32–37^ but extend these approaches by considering an array of composite elements and study their elasto-plastic behaviour. To the best of our knowledge, such a model did not exist. So far, statistical constitutive models were used successfully to explain the cortical bone behaviour at extracellular matrix level with an upscaling to millimetre length scales^28,55^. We downscale and apply it to the compressive behaviour of a single mineralised collagen fibre and, thus, provide the important missing link between the micro- and nanoscale. We verified the assumption of parallelly arranged fibrils within the fibre using synchrotron nanoscale imaging, which confirms the average uniaxial arrangement reported for mineralised turkey leg tendon^27,39,79^ by a 3D measurement, and we consider the model as verified due to its good fit to experiments (Fig. 6 and Table 2).

We saw that the amount of micro- and nanoscale heterogeneity influences the apparent mechanical fibre behaviour (Fig. 7a). When we only allow a very small variation of the micro- and nanomechanical properties, we observed a more immediate overall failure and reduced ductility contrary to experiments^39^. Within the fibre, we have fibrils that can sustain different amount of stresses. During compression, some fibrils fail earlier than others, without leading to an immediate overall fibre failure (apparent system). After the first fibrils fail, load is being distributed among intact fibrils. If all fibrils were mechanically the same (using an average), reaching ultimate values would lead to an abrupt breakdown of the apparent system (brittle failure) neglecting the ductile behaviour at the fibre microscale^39^. Nature prevents an abrupt failure and allows the ductility by scattering the constituents’ mechanical properties in the composite of collagen molecules, mineral particles and extrafibrillar matrix. It corroborates findings that nanoscale heterogeneity can promote energy dissipation in bone^11^. A too high heterogeneity showed a shift towards smaller ultimate strains and increased softening disagreeing with experiments^39^ (Fig. 7a). Thus, a 10-15% micro- and nanomechanical heterogeneity seems to be present in the micropillars. The constant unloading moduli, also in the plastic region, point to an absence of damage, in agreement with our experiments^39^ and independent experimental data from bone extracellular matrix^28^. When reducing ultimate strain values directly (Fig. 7b), we see a rapid decrease in stress and unloading moduli after first fibrils fail. This disagrees with experiments^39^, so that the micro- and nanomechanical heterogeneity also seems more suitable to explain changes in overall ultimate strains.

For the interplay between the intra- and extrafibrillar phases, we observe higher accumulated extrafibrillar plastic strains. The extrafibrillar phase represents the behaviour at fibril-matrix interfaces governed by load shearing between fibrils. This suggests that beyond the elastic regime the ductile microscale behaviour is dominated by shearing and gliding mechanisms within the extrafibrillar matrix, corroborating findings for bone tissue^10,28^. Mineral-to-fibre strain ratios are also smaller than those for fibril-to-fibre including post-yield. This indicates that the organic phase within the fibrils supports the microscale plasticity. This interplay of organic and inorganic phases is fundamental for bone to enable energy dissipation and fracture resistance going beyond the mechanical properties of its individual constituents^2,4,8,9,51^.

One advantage of the model is that simulation results can be directly related to in situ micromechanical testing and SAXS/XRD experiments^39^. Fibril and mineral strains in such combined mechanical and X-ray scattering experiments always represent average values from the probed material inside X-ray beam windows^31,32,39,41^. The measured overall fibril strain might then be the result of fibrils showing very large and very small strains or even no strain at all, e.g. due to strain localisation in a failure zone. This influences reported strain ratios between tissue level and the nanoscale constitutive phases. Our model can simulate this heterogeneous deformation via statistical distributions for the model input and output. Strain ratios between constituents and tissue level are provided in form of histograms (Figs. 6c,d and 4c,d). Their mean values resembles the experimental strain ratios^39^. Thus, applying this to other in situ studies with millimetre sized samples^23,31,32,34,41,43,44^, helps us to understand that overall strain ratios are not only influenced by energy dissipation as discussed in these papers, but also by the heterogeneous deformation of constituents due to an initial micro- and nanomechanical heterogeneity. In our experiment, we also observed that surface roughness led to a gradual fibril recruitment and that only a fraction of the fibrils within the fibre was loaded instantaneously. The load was not distributed equally to the structure. At the yield point, around 10% of the fibrils were still not loaded (Fig. 6) and shortly after all fibrils carried load, first fibrils fail (Supplementary Video 2). This indicates that individual fibrils can fail and cracks can exist while the plastic behaviour is carried by other intact elements and the load is being distributed allowing transitions between subsequent failures of fibrils, similar to explanations for bone tissue^55^. Our SAXS/XRD measurements gave us a mean value for fibril and mineral strains but no distribution within the X-ray beam window. An experimental validation of the simulated distributions may be accessible with volumetric SAXS measurements^80^. These scan regimes were not applicable to our combined testing and most probably would have a detrimental effect on the sample. In the line of thought of a statistically different behaviour of fibrils, one might consider the reported waviness of bone fibrils^37^. A gradual recruitment of such fibrils could be an inherent statistical property of a wavy bone extracellular matrix and an intrinsic property of biological tissue with short range order resulting from loading along various directions during their manufacturing. This hypothesis might help to understand the role of a disordered phase of fibrils for bone mechanics^10^, which not only affect the mentioned synchrotron results. From a structure-mechanics point of view, such a disordered phase could also support the microscale ductility as we have shown in our study based on the influence that a micro- and nanomechanical heterogeneity has on the fibre behaviour.

We can use the compositional information from SRnCT to generalise our model towards other mineralised tissues. The mineralised collagen fibril diameter of $224 \pm 45\,\hbox {nm}$ agrees with values for mineralised tendons reported to be up to 300 nm^1,77^. It is a factor of 1.8 larger than mean values reported for bone^1,81,82^ which can be related to the naturally mineralised tendon being tailored towards tension. A positive correlation between ultimate tensile strength and average collagen fibril diameter exists^83^ with largest diameters found for tissues with the highest tensile loads^84^. Our fibre mineral density of $1.64 \pm 0.16\, \hbox {g/cm}^{3}$ is smaller than the $1.90 \pm 0.07\, \hbox {g/cm}^{3}$^68^ reported for extracellular matrix of human cortical bone. Mineral density shows a direct relation to apparent stiffness in mineralised tissues^8,15,26,85^ and combined with differences in fibril diameter might explain the 45% higher stiffness^28,29^ and 67% higher yield stress in bone^28^.

**Figure 8:**
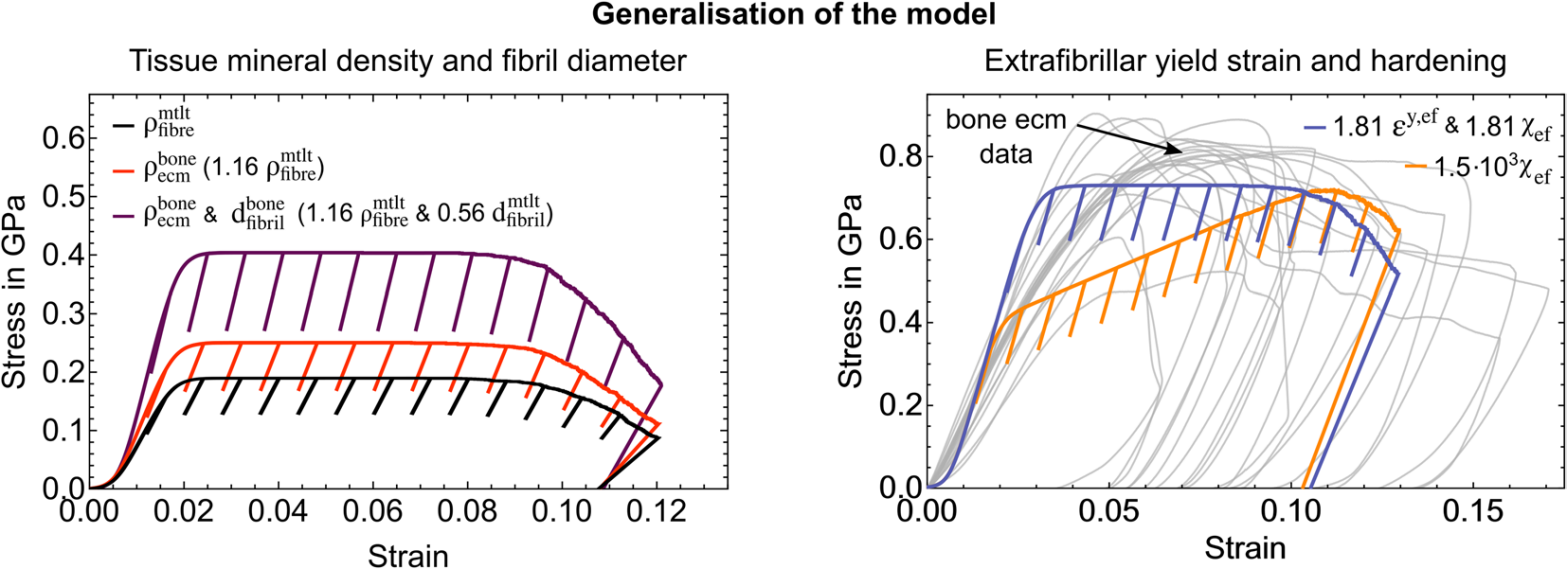
Model generalisation towards cortical bone. (Left) Simulated fibre stress-strain using the higher bone tissue mineral density $\rho$^68^ (*red*) and combined with the smaller bone fibril diameter *d*^1,81,82^ (*magenta*); original composition in *black*. (Right) Simulated fibre stress-strain when accounting for an effective increase of the fibrils surface area per unit mass within the extrafibrillar matrix due to a smaller fibril diameter leading to a change in fibril-matrix interaction. *Blue*: Increased extrafibrillar yield strain and hardening. *Orange*: Sole and very high increase of hardening necessary to reach the bone compressive strength at ecm level^28^. The *grey* curves represent experimental micropillar compression data of bone extracellular matrix^28^. The model was run with a 75% lower surface roughness based on experiments^28^.

We used our model to study these assumptions. The 16% higher bone mineralisation in the nested shear lag model composition, led to a simulated fibre with an apparent stiffness of 20.81 GPa, corresponding to a 32% increase. Yield stress was 0.23 GPa and compressive strength 0.25 GPa (Fig. 8, left, red curve). Compared to an apparent stiffness of $31.16 \pm 6.46\,\hbox {GPa}$^28^, a yield stress of $0.49 \pm 0.1\,\hbox {GPa}$^28^, and a compressive strength between $0.65 \pm 0.02\,\hbox {GPa}$^29^ and $0.75 \pm 0.06\,\hbox {GPa}$^28^ for bone extracellular matrix, we see that the shear lag models with a higher mineralisation alone cannot explain the higher mechanical properties at the microscale. When we combine more and smaller fibrils with the same mechanical properties in a parralel arrangement, the total stress is increased. We use the same standard deviation and fibre diameter while assuming that a change in fibril diameter does not affect their mechanical properties^86^. Thus, an increase in the apparent mechanical properties is reached via the number of fibrils. We approximate this behaviour by implementing the 46% smaller bone fibril diameter^1,77,81,82^ via the corresponding factor of 1.8 in the nested shear lags. The model simulated a fibre with 33.28 GPa apparent stiffness, 0.37 GPa yield stress and 0.41 GPa compressive strength (Fig. 8, left, magenta). Stiffness agrees well with and yield stress is close to results reported for bone extracellular matrix, but not the compressive strength^28,29^. R﻿educing the fibril diameter effectively increases the fibril surface area per unit mass within the extrafibrillar matrix. This makes it more likely that interfibrillar non-covalent crosslinks exist between fibrils and matrix components^83^. These were reported to enable toughening mechanisms^23,38^. Thus, we assume that fibril failure within the matrix is hindered leading to different hardening and/or a higher extrafibrillar yield strain. To approximate the necessary factor between mineralised turkey leg tendon and bone, we calculated fibril surface-to-volume ratios based on fibril diameters. The resulting 1.81 higher ratio for bone was used for hardening and extrafibrillar yield strain (Fig. 8, right). The simulated fibre with a higher mineralisation, smaller fibril diameter, higher hardening modulus and higher extrafibrillar yield strain had a 33.28 GPa stiffness (as for the previous case), around 0.6 GPa yield strain and 0.73 GPa compressive strength (Fig. 8, right, blue). A sole increase in hardening modulus returns a 33.28 GPa stiff fibre with a 0.37 GPa yield stress but a very high hardening value would be necessary to reach the experimental compressive strength^28,29^ (Fig. 8, right, orange). Simulated stress-strain curves also reveal that linear hardening used in the model does not seem to be sensitive enough to simulate different hardening behaviours. Compared to mineralised turkey leg tendon^39^, these simulation results suggest that bone reaches its higher stiffness, yield stress and compressive strength at the extracellular matrix level^28,29^ by a higher mineralisation, different fibril diameter, and consequently, by a change in the fibril-matrix interaction. Using the model, thus, allowed us to explain microscale differences between two types of mineralised tissue^28,29,39^ including independent experimental data.

Although in very good agreement with micro- and nanomechanical experiments, the model was formulated with some assumptions. The role of water in the interaction within the mineralised collagen fibre is not modelled. It has been shown that rehydration has a significant effect on the mechanical properties^37,87–90^. In our model, the bound water is included in the volume fractions of the organic constituents. As there are no data at the fibre level for rehydrated samples, a direct comparison was only possible with dry tested micropillars. A related study where we extended the setup to rehydrated conditions is currently being analysed. In our experiments^39^, we used a quasi-static loading protocol at a displacement rate of 5 nm/s which corresponded to a strain rate of $4\cdot 10^{-4}\, \hbox {s}^{-1}$. Thus, we can neglect viscoelastic effects. To simulate viscoelasticity and loading regimes at higher strain rates, dash pots, parallely arranged with the plastic sliders in the intra- and extrafibrillar phases, could be included. For viscoelasticity, a standard solid model approach could be adopted but the overall model concept would stay the same. Our simulation results for the strain ratios between fibril and fibre show a slightly better agreement than those between mineral and fibre, which might be related to additional deformation or re-orientation mechanisms of the nanocrystals that are not captured by the 1D model. Based on the available uniaxial experiments, we identified the underlying mechanisms with a 1D constitutive model. An extension towards 2D and 3D will be interesting, also from a numerical point of view, given that experimental data are provided. This will also allow us to capture the anisotropic behaviour of the fibre where bimodal statistical distributions might be useful^91^.

Our statistical material model for the mineralised collagen fibre bridges the micro- and nanometre length scales, and explains the elasto-plastic behaviour of bone’s fundamental mechanical building block. We cover elasticity and plasticity, and allow variable micro- and nanomechanical properties for individual model elements of the fibre constitutive phases, which is an advantage to other existing modelling approaches. We study the micro- and nanoscale deformation and failure mechanisms, and investigate the mechanical interplay between organic and inorganic components in mineralised tissues while considering the micro- and nanoscale heterogeneity. Our findings add an important contribution to combine models at different hierarchical levels of bone aiming to simulate its elasto-plasticity from the molecular up to the macroscopic length scale. By deducing information on changes of bone diseases, especially at the molecular level, such models can be a cornerstone to mitigate challenges imposed by ageing societies. The knowledge at these small length scales is also fundamental to mimic the true behaviour of biological tissues. It guides us in the design and development of future bio-inspired materials.

## Methods

In the following, detailed information is presented on: (1) the analytical mathematical framework of the two nested shear lag models including the connection to a single elasto-plastic rheological element (“Two nested shear lag models for a mineralised collagen fibre” section); (2) the analytical mathematical framework of a single rheological unit for the fibre composite with two inelastic strain mechanisms ("Analytical mathematical framework for a single rheological element" section); (3) the parallel arrangement of several rheological elements with a statistical description ot material properties ("Statistical material properties" section); (4) the measurements and analysis of SRnCT to determine the tissue mineral density, the fibril diameter, the local 3D fibril orientation and the surface roughness of the micropillars (“SRnCT of the ultrastructure of a mineralised collagen fibre” section); and (5) a summary of the used model parameters (“Model parameters” section together with Table 2).

### Two nested shear lag models for a mineralised collagen fibre

Based on existing shear lag models^26,47,58–60^, we derived the effective apparent Young’s modulus of the mineralised collagen fibril (mineral-collagen composite $=$ mc) $\epsilon ^{fibril} = \epsilon ^{mc}$ where especially the works by Gao^60^ and Yao and Gao^47^ were used for the multilevel approach.

Regarding composition, at the fibre level bone tissue is divided in mineralised collagen fibrils (mc) and extrafibrillar matrix (ef) that are composed of mineral (min)/collagen (col), respectively non-collagenous proteins (ncp) (Fig. 2, left). The corresponding volume fractions are related as follows:

1 $$\begin{aligned} \phi _{mc} +\phi _{ef}=1 \qquad \phi _{min}+\phi _{col}+\phi _{ncp}=1 \end{aligned}.$$

The tissue density is related to the constituent densities by (value from Results section "Tissue mineral density within a fibre"):

2 $$\begin{aligned} \rho = \phi _{min}\;\rho _{min}+\phi _{col}\;\rho _{col} +\phi _{ncp}\;\rho _{ncp}=1.64 \; \text {g/cm}^{3} \end{aligned},$$

with^9,92,93^:

3 $$\begin{aligned} \rho _{min}=2.90 \; \text {g/cm}^{3} \qquad \rho _{col}=\rho _{ncp}=1.13 \; \text {g/cm}^{3} \end{aligned}.$$

The shear lag model^47,59,60^ applied to the mineralised collagen fibril provides their apparent elastic modulus, which represents the effective stiffness of the series arrangement of intra- and extrafibrillar phases:

4 $$\begin{aligned} \epsilon ^{mc} = \left( \frac{1}{\phi _{min}~\epsilon ^{min}} + \frac{4~(1-\phi _{min})}{\phi _{min}^2 \gamma _{min}^2\mu ^{col}}\right) ^{-1} \end{aligned},$$

where $\phi _{min}$ is the mineral volume fraction, $\epsilon ^{min}$ the mineral particle Young’s modulus, $\gamma _{min}$ the mineral particle aspect ratio and $\mu ^{col}$ the collagen shear modulus.

Assuming that the axial stress in the mineralised collagen fibril is carried by the mineral (stiff part in the compliant matrix)^47,59,60^, the strain of the intrafibrillar mineral can be calculated by:

5 $$\begin{aligned} \varepsilon ^{min,mc} =\varepsilon ^{e,min}=\frac{\sigma ^{mc}}{\phi _{min}\epsilon ^{min}} \end{aligned}.$$

Similarly, the apparent mineralised collagen fibre Young’s modulus $\epsilon$ is given by^47,59,60^:

6 $$\begin{aligned} \epsilon = \left( \frac{1}{\phi _{mc}~\epsilon ^{mc}} + \frac{4~(1-\phi _{mc})}{\phi _{mc}^2~\gamma _{mc}^2\mu ^{ef}}\right) ^{-1} \end{aligned},$$

where $\phi _{mc}$ is the mineralised collagen fibril volume fraction, $\epsilon ^{mc}$ the mineralised collagen fibril Young’s modulus, $\gamma _{mc}$ the fibril aspect ratio and $\mu ^{ef}$ the extrafibrillar matrix shear modulus.

Assuming again that the axial stress is carried by the stiffer part, here the mineralised collagen fibril within the extrafibrillar matrix, the elastic strain of the fibril can be expressed by:

7 $$\begin{aligned} \varepsilon ^{e,mc} =\frac{\sigma ^{mc}}{\epsilon ^{mc}}=\frac{\sigma }{\phi _{mc}\epsilon ^{mc}} \end{aligned}.$$

Using the decomposition of the mineralised collagen fibril strain into an elastic and a plastic part (Methods section “Elasto-plastic constitutive model for a fibril array” and Eq. (11)), the ratio between the apparent strain of the fibril and the total strain can then be calculated via:

8 $$\begin{aligned} \frac{\varepsilon ^{mc}}{\varepsilon } = \frac{1}{{\varepsilon }}\left( \frac{\sigma }{\phi _{mc}\epsilon ^{mc}} + \varepsilon ^{p,mc}\right) \end{aligned}.$$

With Eqs. (5) and (8), we determine the strain ratio between the mineral particles of the fibril and the total strain:

9 $$\begin{aligned} \frac{\varepsilon ^{min}}{\varepsilon } = \frac{1}{{\varepsilon }} \frac{\sigma }{\phi _{mc}\phi _{min}\epsilon ^{min}} \end{aligned}.$$

### Elasto-plastic constitutive model for a fibril array

The primary underlying idea of the model is a statistical distribution (Methods section "Statistical material properties") of rheological units (Methods section "Analytical mathematical framework for a single rheological element") that are each composed of a mineralised collagen (mc) fibril embedded in an extrafibrillar (ef) matrix. The statistical description pertains to both their material properties as well as their recruitment in the volume of the micropillar.

#### Analytical mathematical framework for a single rheological element

A single rheological unit consists of an elastic spring and two plastic sliders that account for the inelastic strains in the intra- and extrafibrillar phases. The model, thus, simulates the failure at the mineral-collagen (mc) interface and fibril-matrix (ef) interface, respectively (Fig. 2). The elasto-plastic rheological model is rate-independent and based on the thermodynamics of irreversible processes^28,54,55,94,95^. The series arrangement of both phases allows different strains and a deformation localisation in each of them^31,33,39,41^. Accordingly, an additive decomposition of the total strain of the fibre into an intra- and an extrafibrillar strain is assumed^62^:

10 $$\begin{aligned} \varepsilon = \varepsilon ^{mc}+\varepsilon ^{ef} \end{aligned}.$$

Since inelastic strains accumulate in the intra- and extrafibrillar phases, the plastic deformation is split into $\varepsilon ^{p,mc}$ and $\varepsilon ^{p,ef}$:

11 $$\begin{aligned} \varepsilon =\varepsilon ^{e} + \varepsilon ^{p,mc} + \varepsilon ^{p,ef} \end{aligned}.$$

For conciseness, the elastic strain of the two deformation mechanisms $\varepsilon ^e$ is pooled. We use a piecewise definition of the Helmholtz free energy where its formulation depends on whether the strain in the intra- or extrafibrillar phase has reached their ultimate value (Eqs. (25) and (26)). We consider an isothermal process and the Helmholtz free energy $\psi$ is then given by^94,95^:

12 $$\begin{aligned} \psi (\varepsilon , \varepsilon ^{p,mc}, \varepsilon ^{p,ef})=\frac{1}{2}~\epsilon (\varepsilon - \varepsilon ^{p,mc} - \varepsilon ^{p,ef})^2 \end{aligned},$$

where $\epsilon$ denotes the apparent (effective) Young’s modulus of the fibre calculated via the two nested shear lag models (Eq. (6) in Methods section “Two nested shear lag models for a mineralised collagen fibre”). The total stress is determined as the partial derivative of the free energy $\psi$ with respect to the total strain $\varepsilon$^94^ and is then given by:

13 $$\begin{aligned} \sigma = \frac{\partial \psi }{\partial \varepsilon } = \epsilon (\varepsilon - \varepsilon ^{p,mc} - \varepsilon ^{p,ef}) \end{aligned}.$$

The plastic stresses $\sigma ^{p,mc}$ and $\sigma ^{p,ef}$ are conjugated to the plastic strains $\varepsilon ^{p,mc}$ and $\varepsilon ^{p,ef}$, respectively, and are equivalent to the total stress. For isothermal processes, the dissipation $\Phi$ is determined by^53,55,94^:

14 $$\begin{aligned} \Phi = \sigma {\dot{\varepsilon }} -{\dot{\psi }} =\sigma ^{p,mc} {\dot{\varepsilon }}^{p,mc} + \sigma ^{p,ef} {\dot{\varepsilon }}^{p,ef} \ge 0 \end{aligned}.$$

This local formulation of the Clausius-Duhem inequality expresses the second law of thermodynamics and must imperatively be verified by the flow of the plastic strains. In rate-independent elasto-plastic models, the evolution of the plastic strains (Figs. 1 and 2) can be defined via yield functions of the plastic stresses^28,54,95^. The intra- and extrafibrillar yield criteria are then defined by:

15 $$\begin{aligned} f(\sigma ^{p,mc})&:= |{\sigma ^{p,mc}}| - h^{mc} = |{\sigma ^{p,mc}}| - \epsilon \varepsilon ^{y,mc} - \chi ^{mc} \alpha ^{mc} \end{aligned},$$

16 $$\begin{aligned} f(\sigma ^{p,ef})&:= |{\sigma ^{p,ef}}| - h^{ef} = |{\sigma ^{p,ef}}| - \epsilon \varepsilon ^{y,ef} - \chi ^{ef} \alpha ^{ef} \end{aligned}.$$

$\varepsilon ^{y,mc}$ and $\varepsilon ^{y,ef}$ denote the yield strains, and $\chi ^{mc}$ and $\chi ^{ef}$ the hardening moduli for the mineralised collagen fibril and the extrafibrillar matrix, respectively (Table 2 in Methods section “Model parameters”). $\alpha ^{mc}$ and $\alpha ^{ef}$ denote the accumulated plastic strains in the two phases:

17 $$\begin{aligned} \alpha ^{mc}&= \int _0^t |{{\dot{\varepsilon }}^{p,mc}}|~d\tau \end{aligned},$$

18 $$\begin{aligned} \alpha ^{ef}&= \int _0^t |{{\dot{\varepsilon }}^{p,ef}}|~d\tau \end{aligned}.$$

The flow rules can then be defined via the yield functions of the plastic stresses (Eq. (15) and 16):

19 $$\begin{aligned} {\dot{\varepsilon }}^{p,mc}&= {\dot{\lambda }}_L^{mc} \frac{\partial f}{\partial \sigma ^{p,mc}} = {\dot{\lambda }}_L^{mc} \frac{\sigma ^{p,mc}}{|\sigma ^{p,mc}|} \end{aligned},$$

20 $$\begin{aligned} {\dot{\varepsilon }}^{p,ef}&= {\dot{\lambda }}_L^{ef} \frac{\partial f}{\partial \sigma ^{p,ef}} = {\dot{\lambda }}_L^{ef} \frac{\sigma ^{p,ef}}{|\sigma ^{p,ef}|} \end{aligned}.$$

Since the sign of each plastic strain is the same as the conjugated plastic stress, the Clausius–Duhem inequality is fulfilled at all times. The Karush–Kuhn–Tucker conditions^28,95,96^ distinguish the elastic ($f(\sigma ^{p,mc}) < 0, {\dot{\lambda }}_L^{mc}=0$) from the plastic ($f(\sigma ^{p,mc}) = 0, {\dot{\lambda }}_L^{mc}>0$) regime:

21 $$\begin{aligned} f(\sigma ^{p,mc})&\le 0, \qquad {\dot{\lambda }}_L^{mc} \ge 0, \qquad {\dot{\lambda }}_L^{mc} f(\sigma ^{p,mc}) = 0 \end{aligned},$$

22 $$\begin{aligned} f(\sigma ^{p,ef})&\le 0, \qquad {\dot{\lambda }}_L^{ef} \ge 0, \qquad {\dot{\lambda }}_L^{ef} f(\sigma ^{p,ef}) = 0 \end{aligned}.$$

The plastic stresses for the intra- and extrafibrillar phases can then be written in relation to the rate of the plastic strains:

23 $$\begin{aligned} \sigma ^{p,mc}&= {\left\{ \begin{array}{ll} -\epsilon \varepsilon ^{y,mc} - \chi ^{mc} \alpha ^{mc}, &{} {\dot{\varepsilon }}^{p,mc} < 0\\ \left[ -\epsilon \varepsilon ^{y,mc} - \chi ^{mc} \alpha ^{mc};~\epsilon \varepsilon ^{y,mc} + \chi ^{mc} \alpha ^{mc}\right] , &{} {\dot{\varepsilon }}^{p,mc} = 0\\ \epsilon \varepsilon ^{y,mc} + \chi ^{mc} \alpha ^{mc}, &{} {\dot{\varepsilon }}^{p,mc} > 0 \end{array}\right. } \end{aligned},$$

24 $$\begin{aligned} \sigma ^{p,ef}&= {\left\{ \begin{array}{ll} -\epsilon \varepsilon ^{y,ef} - \chi ^{ef} \alpha ^{ef}, &{} ~~~{\dot{\varepsilon }}^{p,ef} < 0\\ \left[ -\epsilon \varepsilon ^{y,ef} - \chi ^{ef} \alpha ^{ef};~\epsilon \varepsilon ^{y,ef} + \chi ^{ef} \alpha ^{ef}\right] , &{} ~~~{\dot{\varepsilon }}^{p,ef} = 0\\ \epsilon \varepsilon ^{y,ef} + \chi ^{ef} \alpha ^{ef}, &{} ~~~{\dot{\varepsilon }}^{p,ef} > 0 \end{array}\right. } \end{aligned}.$$

However,

25 $$\begin{aligned} \sigma ^{p,mc}= & {} \ 0, \qquad \varepsilon ^{p,mc} \notin \left[ -\varepsilon ^{p,mc,ult},\varepsilon ^{p,mc,ult}\right] \end{aligned},$$

26 $$\begin{aligned} \sigma ^{p,ef}= & {} \ 0, \qquad \varepsilon ^{p,ef} \ \notin \left[ -\varepsilon ^{p,ef,ult},\varepsilon ^{p,ef,ult}\right] \end{aligned},$$

which expresses that the sliders are deactivated when they reach their ultimate strains and that both total stress $\sigma =0$ and dissipation $\Phi =0$ whenever one of the sliders fails.

#### Statistical material properties

The uniaxial fibril array within the extrafibrillar matrix is represented by N elasto-plastic rheological elements (Figs. 1 and 2) where the model parameters (Table 2) represent an average mineralised collagen fibre with mineralised collagen fibrils and extrafibrillar matrix, which we then redistribute onto our parallel arrangement. Values of the single elements are then calculated based on the total number of model elements following the mathematical approximation also presented in previous works^28^. To include and study the heterogeneity of mechanical properties in biological tissues^2,8,9^, we used statistical distributions. Mathematically, this is allowed since we work with a parallel arrangement of fibrils embedded in an extrafibrillar matrix, where the total stress $\sigma _{tot}$ corresponds to the sum of the partial stresses of these fibrils (rheological elements) (Figs. 1, 2, and Supplementary Sect. S1). We assumed a normal distribution^28,55^ for a variable *X*, so that $P(X) \sim {\mathcal {N}}( \mu , \varsigma ^2)$ with $\mu$ as the mean and $\varsigma$ as the standard deviation of *X* for *n* rheological elements. Thus, normal distributions of a variable X were calculated via $P(X) = \frac{1}{\varsigma \sqrt{2\pi }}~e^{-\frac{\left( x-\mu \right) ^2}{2\varsigma ^2}}$ for all mechanical parameters such as stiffness values, yield strains and ultimate strains as well as dependent quantities (Table 2 in section "Model parameters"). Where accessible, mean values were taken from micropillar compression at the mineralised collagen fibre level where results were normally distributed^39^. Using a normal distribution for the statistical description and a base value of 15% for its standard deviation $\varsigma$ is further motivated by our own experimental results presented in this study including the tissue mineral density" and fibril diameter (Results sections "Tissue mineral density within a fibre" and "Fibril diameter and number of rheological elements") as well as independently reported mechanical values from micro- and nanopillar compression^10,28,29^, micro- and nanoindentation^10,97,98^, and mineralisation measurements^98,99^. In addition, we used a range of 1% to 30% of statistical variation during the sensitivity analysis to study the influence of the micro- and nanomechanical heterogeneity on the fibre behaviour (Fig. 7). By using distributions for the input variables, we cover a certain amount of scattering of the model input and get a distribution of output values including the strain ratios calculated based on the shear lag models between mineral particles, collagen molecules, mineralised collagen fibrils and mineralised collagen fibre ($P(\varepsilon _{mc}/\varepsilon )_n$, $P(\varepsilon _{ef}/\varepsilon )_n$ and $P(\varepsilon _{mc}/\varepsilon _{ef})_n$).

### SRnCT of the ultrastructure of a mineralised collagen fibre

SRnCT^68^ with a voxel size of (20 nm)$^{3}$ was used to determine the tissue mineral density (Results section "Tissue mineral density within a fibre", Methods section "Tissue mineral density within a fibre" and Supplementary Sect. S3), the fibril diameter (Results section "Fibril diameter and number of rheological elements" and Methods section "Fibril diameter, packing density and number of rheological units") and the 3D local orientation of the mineralised collagen fibrils of the fibre micropillars used in previous experiments^39^. These are material dependent parameters to inform the model composition. The surface roughness was determined to account for an experimental artefact that led to a gradual fibril recruitment during the compression. This parameter is material independent and related to the fabrication process^39^. Experimental data were obtained at beamline ID16A of the European Synchrotron Radiation Facility (ESRF). The main advantage of SRnCT compared to conventional attenuation based tomography^100,101^ is the high sensitivity for density variations in a probed sample since even light organic elements cause a phase shift of the X-ray beam. We can detect small structural changes via small variations in mass density distributions and extract an absolute value for the local tissue mineral density at the micro- and sub-microscale^64–68^. To obtain each 3D image, four tomographic scans were acquired with the sample positioned at different distances with respect to the X-ray focus and the detector. For each tomographic scan, 1600 angular projections were recorded with an exposure time of 0.2 s at an X-ray beam energy of 17.05 keV ($\lambda \simeq 0.73 \,\mathring{A}$), with a monochromaticity of 1%. The projections were recorded on a 2Kx2K lens coupled FreLoN CCD detector and the field of view was $40\, \upmu \hbox {m}$.

The samples were mounted on Huber pins (HUBER Diffraktionstechnik, Rimsting, Germany) and placed on a closed-loop nanopositioning stage inside a vacuum chamber for imaging. The four projections recorded at different focus-to-sample-to-detector distances corresponding to a given rotation angle were combined together in a phase retrieval algorithm based on regularised contrast transfer function (CTF). The resulting phase maps were used for tomographic reconstruction based on filtered back-projection^68^. The obtained 3D images give the distribution of the refractive index decrement $\delta _{dec}$ (Supplementary Sect. S3) inside the sample to determine the mass density distribution. These reconstructed images were used for different structural analyses for which we have selected VOIs with a width and length of 8.5 $\upmu \hbox {m}$ x 8.5 $\upmu \hbox {m}$ (ROI 1 in Fig. 3) and a variable depth based on the analyses. Supplementary Sect. 3 provides detailed information on the SRnCT technique and reconstruction procedure.

#### Tissue mineral density within a fibre

The local mass density value $\rho$ in $g/cm^{3}$ of each voxel can be determined from the reconstructed refractive index decrement $\delta _{dec}$ of the SRnCT images (Supplementary Sect. S3). $\delta _{dec}$ can be written based on the Guinier approximation^72^: $\delta _{dec} = 2.72 \cdot 10^{-6}~\frac{Z}{M}~\rho ~\lambda ^2$. By assuming the factor of $M/Z \approx 2$, the refractive index is thus given by $\delta _{dec} \cong 1.3\cdot 10^{-6}~\rho ~\lambda ^2$ where $[\lambda ] = \mathring{A}$. The SRnCT reconstruction provides values for $\omega = - \frac{2\pi }{\lambda }~\delta _{dec}$ with $[\omega ] = \hbox {cm}^{-1}$. Using the Guinier approximation, we get $\rho = - \frac{1}{2\pi }~\frac{\omega }{1.3~\lambda } \cdot 10^{-2}$ where $\omega$ and $\lambda$ are in cm^-1^. Thus, the refractive index value of each voxel in the reconstructed SRnCT images can be converted to a mass density $\rho$, providing real values of the local 3D mass density distribution. Grey values related to mineralised tissue and the background signal were analysed via probability density distributions (Supplementary Fig. S7). Corresponding mean values of both regions were identified by Gaussian fits and the full width at half maximum (FWHM). Initial fitting values for peak position and variance for the Gaussian distributions were identified via a peak finding algorithm. The standard deviation $\varsigma$ of the tissue mineral density value was determined via the FWHM of the Gaussian fit as $\varsigma = \frac{\text {FWHM}}{2\sqrt{2~ln(2)}}$. Data analysis was done with custom written codes in R (v3.6.2)^73^ and Python (v2.7)^78^ with pre-processing steps in Fiji (imageJ v1.53c)^70^. The raw $\omega$-values needed to be corrected since the micropillars could be larger than the X-ray beam, introducing local tomography artefacts. As a first approximation, the correction may be achieved by shifting the grey levels by an offset as reported in^68^. There, pores or lacunae in the individual volumes of interests were used to estimate this offset. In our study, the background signal next to the base of the micropillar was used for the global offset calculation since it showed the best comparability with the reported approach (Supplementary Sect. S3.1 and Figs. S7, S8). A selection was necessary since three different areas related to the background were identified where the X-ray beam did not directly interact with the sample (Supplementary Sect. S3.1 and Fig. S8). These regions showed different grey value distributions. VOIs with a depth of 100-150 slices, depending on the free space next to the micropillar, were selected to determine the mean grey value as the peak of the probability density distribution (Supplementary Fig. S7). This served as the global offset value.

#### Fibril diameter, packing density and number of rheological units

The diameter and number of mineralised collagen fibrils within a fibre of $6\,\upmu \hbox {m}$ in diameter^39^ were determined via line profiles from sagittal views of reconstructed SRnCT images (Fig. 3b). First, a region of interest (ROI 1) of $10 \,\upmu \hbox {m}$ x $10\, \upmu \hbox {m}$ was chosen around the micropillar area (Fig. 3a). Within ROI 1, a smaller region (ROI 2) of around $5.5\,\upmu \hbox {m}$ × $5.5\, \upmu \hbox {m}$ was selected from the undeformed micropillar area (Fig. 3b) where brighter regions of the inverted image are related to fibrils, darker regions to extrafibrillar matrix (Fig. 3c). To increase the signal-to-noise ratio, an adaptive edge-sensitive speckle reducing anisotropic diffusion was used that enhances images that show a signal-dependent, spatially correlated multiplicative noise^69^ (Fig. 3d). Parameters for the diffusion filter, implemented in Fiji (imageJ v1.53c)^70^ slice by slice, were: diffusion coefficient threshold 0.1, mean square error limit 0.01, maximum of iterations 60, initial coefficient of variation 1.0, coefficient of variation decay rate 0.1667, and time step 0.05. The preservation of structural image features after the diffusion is visible in Fig. 3. Within the filtered images, three to five even smaller regions of interest (ROI 3) of around $2\,\upmu \hbox {m}$ x $2\,\upmu \hbox {m}$ per sample were chosen (Fig. 3). Three to five neighbouring fibrils were accessible for the analysis in ROI 3 and line profiles were extracted. The profiles provided mean grey-values for all pixels along each column of ROI 3. Signal width and periodicity related to fibrils (including extrafibrillar matrix) were analysed via local minima and Fast Fourier Transform (FFT), respectively. Since fibril and extrafibrillar matrix are seen as one entity in the model, due to their series arrangement, this approach is justified. Both approaches were implemented in R (V3.6.2)^73^. 23 line profiles were analysed with a total of 81 fibrils. Mean values and standard deviations for the periodicity in the line profiles and the signal widths were calculated (Fig. 3 and Results section "Fibril diameter and number of rheological elements"). The average of both analyses were used as the final result with the error calculated via their variances^102,103^. The fibrils appeared as round and densely packed within the fibre (Fig. 3b, Supplementary Fig. S8, left) and a closest packing of circles in a circle was assumed. The packing density was then calculated based on a local optimisation algorithm of solving circular packing problems^104^. The identified value *d* served to determine the number of rheological elements present for the average diameter of the tested fibres^39^.

#### Local 3D fibril orientation

Average orientation values for the mineralised collagen fibrils and mineral particles within uncompressed micropillars were reported from SAXS and XRD measurements of mineralised turkey leg tendon^39^. Small deviations from the longitudinal axis for both components were measured, $5.76 \pm 4.99^{\circ }$ for fibrils and $4.78 \pm 3.81^{\circ }$ for mineral particles (X-ray beam window of $5.5\,\upmu \hbox {m}$ x $7.0\,\upmu \hbox {m}$). These did not include out of plane orientations. The local fibril 3D orientation within tested micropillars and the sample volume below (Fig. 1) were, thus, analysed with an autocorrelation-based approach^74^. The autocorrelation function $ACF = \left| F_{d}^{-1}\left( F_d(I)~\text {conj}(F_d(I))\right) \right|$ can be used to quantify the orientational dependence of periodicity in the SRnCT reconstructions^66,74^, where $F_d$ denotes the discrete Fourier transform and $F_d^{-1}$ the discrete inverse Fourier transform of the image *I*. Details on the 3D ACF, and how to determine orientation and degree of anisotropy (DA) can be found in Wald et al.^66^ and Varga et al. (2013)^74^. Pre-processing included a slice-wise scaling of the greyscale values to match the mean intensities of each xy slice. This was necessary due to large intensity deviations within reconstructed images along the z-axis. The sample domain excluding larger failure zones was identified by thresholding and morphological operations by which the corresponding mask was generated. The local orientation was analysed in small cubical sub-regions whose edge length (60 voxels $= 1.2\,\upmu \hbox {m}$) was identified in a convergence study to be small enough to avoid excessive averaging and large enough to achieve sufficient input signal quality to the 3D ACF^74^. The isotropic analysis grid had a spacing of $0.6\,\upmu \hbox {m}$, the adjacent analysis sub-regions were overlapping with half of their volumes. In a given sub-region, the fibril orientation was then evaluated as the major semi-axis direction of the ellipsoidal fit of the thresholded 3D ACF, whose principal components represent the fabric tensor^66^. Based on the three Eigenvectors, the largest ACF Eigenvalue and the degree of anisotropy (DA)^66^ can be calculated, which was then used to quantify the fibril orientation angle^74^. To avoid confounding effects of large failure zones of the compressed micropillars, only sub-volumes located completely within the sample domain were analysed. The DA of the ACF, i.e. the ratio of the largest to the smallest Eigenvalues, was used as the fidelity measure of the evaluated orientation following Varga et al.^74^.

#### Surface roughness and fibril recruitment

**Figure 9:**
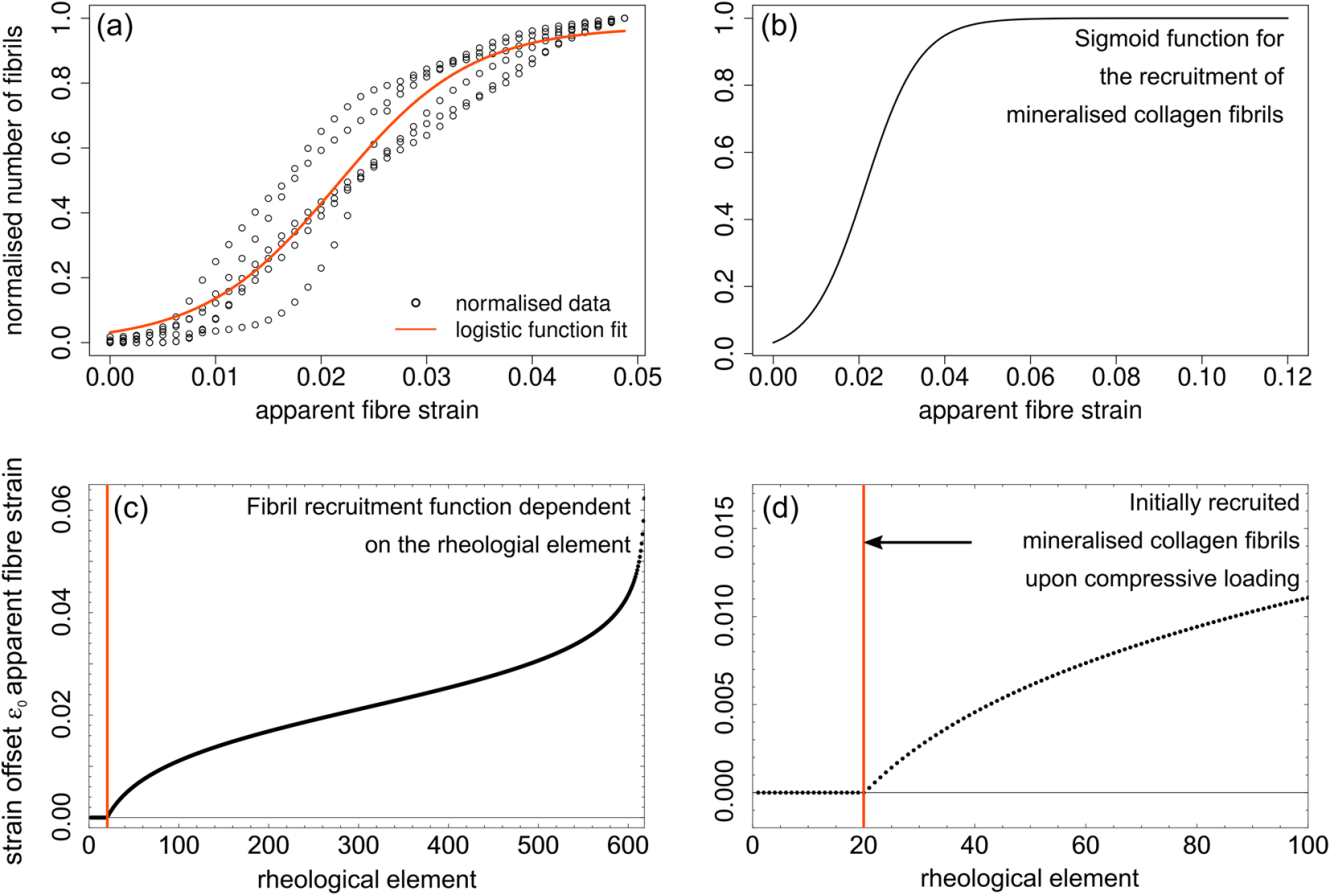
Details to derive the non-linear function for the gradual fibril recruitment. Based on cross-sectional SRnCT slices area fractions relative to the point of full contact between flat punch and micropillar were calculated (Fig. 5). (**a**) Using the fibril diameter and assuming densest packing, a normalised number of fibrils along the loading direction was calculated and fitted with a non-linear function (sigmoid function). (**b**) Extension to cover the full loading range with full contact between 0.05 and 0.12 fibre strain. A plateau is reached after all mineralised collagen fibrils (rheological elements) have been recruited. (**c**) Function curve for the strain offsets $\varepsilon _{0n}$ for every rheological element *n*. (**d**) 20 rheological elements are initially recruited.

To relate the grey-value areas of the micropillar in SRnCT slices (Fig. 5c), areas in pixels were converted to $\hbox {nm}^{2}$ ($\hbox {A}_{1}$, $\hbox {A}_{2}$ ... $\hbox {A}_{{\text{full}}}$) using the voxel size of (20 nm)$^{3}$ and normalised with respect to the full contact area $\hbox {A}_{{\text{full}}}$. Based on the fibril diameter, we determined the corresponding normalised number of fibrils and elements, respectively (Figs. 5d and 9a). A non-linear fit showed that the gradual fibril recruitment can be described with a sigmoid function $\xi (\varepsilon )$ (Eq. (27) and Fig. 9a,b), which was implemented in the statistical constitutive model:

27 $$\begin{aligned} \xi (\varepsilon ) = \frac{1}{1 + e^\frac{-\left( \varepsilon -\mu \right) }{\beta }} = \frac{A_k(\varepsilon )}{A_K} \simeq \frac{n(\varepsilon )}{N} \end{aligned}.$$

$\varepsilon$ denotes the total strain where the reference length is the maximum displacement until full contact. $\frac{A_k(\varepsilon )}{A_K}$ is the area fraction in contact with the flat punch at a given displacement relative to the total surface area $A_{K}$ at full contact. $\mu$ and $\beta$ are the mean value and the shape parameter of the sigmoid function fit. Based on the fibril diameter, $\frac{A_k(\varepsilon )}{A_K}$ can be expressed as a normalised number of fibrils and, thus, rheological model elements, i.e. $\frac{n(\varepsilon )}{N}$ where *N* is their maximum number in the parallel arrangement (Figs. 1, 9, and Results section “Statistical elasto-plastic fibre model at micro- and nanoscale”). The strain offset of each rheological element (n) (embedded fibril) can then be calculated using Eq. (27) (Fig. 9c,d) via:

28 $$\begin{aligned} \varepsilon _{0n} = -\beta ~ln\left( \frac{N}{n}-1\right) +\mu \end{aligned}.$$

Total strains $\varepsilon _n$ are then corrected for each element *n* by the element-specific offset strain $\varepsilon _{0n}$:

29 $$\begin{aligned} \varepsilon _n= \varepsilon _n - \varepsilon _{0n} \qquad \text {if} \qquad \varepsilon _n \ge \varepsilon _{0n} \end{aligned}.$$

Since $\varepsilon _{0n}$ is a constant for a rheological element *n*, the relationships for the analytical mathematical framework (Methods section "Analytical mathematical framework for a single rheological element") and the numerical algorithm (Supplementary Sect. S1) of the model stay valid. The intersection point of the curve with the x-axis (Figures 9a,b) corresponds to the offset of the sigmoid function for the normalised number of fibrils (Fig. 9c). It represents the number of fibrils that are recruited instantaneously when the fibre is compressed. The sigmoid function fit provided values of $\mu = 0.0215$ and $\beta = 0.00635$, which were used in the simulation.

### Model parameters

**Table 2:** Model parameters for a mineralised collagen fibre. The table lists the compositional parameters of the fibre (first section of the table) to calculate fibril and fibre elasticity (second section of the table) via two nested shear lag models. Plasticity calculations were done based on the elasto-plastic rheological elements (third section of the table). The number of rheological elements equals the number of fibrils. Own experimental values were taken where possible and referenced to the corresponding sections. Additional values were taken from the literature.

Model parameter	Variable	Value	Std	Source	Use in model
Fibril volume fraction in fibre	$\phi _{mc}$	0.86	–	Sections "Fibril diameter and number of rheological elements" and "Fibril diameter, packing density and number of rheological units"	Compositional parameters
Number of fibrils	$n_{ele}$	618	–	Sections "Fibril diameter and number of rheological elements" and "Fibril diameter, packing density and number of rheological units"	
Mineral volume fraction	$\phi _{min}$	0.29	–	^15,51,59,77,99^	
NCP volume fraction	$\phi _{ncp}$	0.14	–	^50^	
Fibril aspect ratio	$\gamma _{fibril}$	200	–	^1^	
Mineral particles aspect ratio	$\gamma _{min}$	25	–	^1,33,105,106^	
Mineral Young’s modulus	$\epsilon _{min}$	114 GPa	15%	^1,105,107,108^	
Collagen shear modulus	$\mu _{col}$	1.58 GPa	15%	^1,107^	
EFM shear modulus	$\mu _{ef}$	0.003 GPa	15%	^15^	
Fibril Young’s modulus	$\epsilon ^{mc}$	20.36 GPa	15%	Section “Two nested shear lag models for a mineralised collagen fibre”	Elasticity
Fibre Young’s modulus	$\epsilon$	15.82 GPa	15%	Section “Two nested shear lag models for a mineralised collagen fibre”	
Fibril yield strain	$\varepsilon ^{y,mc}$	0.028	15%	^33,63^	Plasticity
EFM yield strain	$\varepsilon ^{y,ef}$	0.012	15%	using $\varepsilon ^{y,mc}$,^39^ and Eq. (10)	
Fibril hardening modulus	$\chi _{mc}$	0.020 GPa	15%	0.1% of $\epsilon ^{mc}$^28^	
EFM hardening modulus	$\chi _{ef}$	$0.03\cdot 10^{-4}~\hbox {GPa}$	15%	0.1% of $\epsilon _{ef}$ (via $\mu _{ef}$)^28^	
Fibril ultimate strain	$\varepsilon ^{p,mc,ult}$	$\varepsilon ^{p,mc,ult}\cdot 10^{2}$	15%	EFM failure assumed^37,39,109^	
EFM ultimate strain	$\varepsilon ^{p,ef,ult}$	0.09	15%	Using^39^, $\varepsilon ^{p,mc,ult}$ (Section “Model parameters”)	

Std: standard deviation for statistically distributed mechanical properties, NCP: non-collageneous proteins (includes the volume fraction occupied by water when the tissue is wet), EFM: extrafibrillar matrix, mc: mineral-collagen composite (= mineralised collagen fibril), ef: extrafibrillar.

The presented model is valid for the compressive behaviour of a mineralised collagen fibre under dry conditions for which experimental data exists^39^. In our model, the bound water is included in the volume fractions of the organic constituents. Details can be found at the end of the "Discussion and conclusion" section where we mention the limitations of the model. In the Results section “Statistical elasto-plastic fibre model at micro- and nanoscale”, as well as Methods sections “Two nested shear lag models for a mineralised collagen fibre” and “Elasto-plastic constitutive model for a fibril array”, we described the model concepts. Table 2 follows the same structure. We first use the two nested shear lag models to calculate elasticity  . Volume fractions of fibrils (see Results section "Fibril diameter and number of rheological elements" and Methods section "Fibril diameter, packing density and number of rheological units"), mineral^15,50,51,59,77^ and non-collagenous proteins (ncp)^50^ were used based on both the presented SRnCT analysis and literature values. Aspect ratios for fibrils^1^, and mineral particles^1,33,105,106^ as well as the mineral Young’s modulus^1,105,107,108^ and collagen shear modulus^1,107^ were informed by the literature. The shear lag results were used as input for the elasto-plastic rheological units. Additional rheological model parameters were informed by our experiments and, where not accessible, by the literature (see references in Table 2). The number of rheological elements was identified via the SRnCT analysis. For the ultimate strain, the initial failure was assumed to occur in the extrafibrillar matrix with no complete debonding of intrafibrillar mineral and collagen^37,39,109^. The fibril yield strain is based on reported experiments^33,63^ and the extrafibrillar yield strain then resulted from the experimental fibre yield strain^39^ and the series arrangement of our model (Eq. (10) in “Methods”). Hardening moduli were assumed to be 0.1% of the elastic moduli^28^. For all mechanical properties, we used a statistical distribution with a standard deviation of 15%. Further details are provided in Results section “Statistical elasto-plastic fibre model at micro- and nanoscale” and Methods section "Statistical material properties".

### Extraction of mineralised collagen fibre micropillars

For the comparison between experiments and model outcome, experimental results from our previous SAXS/XRD and micropillar compression study were used^39^ where a detailed description of sample preparation and experimental procedures can be found. To summarise. For those experiments, micropillars (diameter: $6.1\,\upmu \hbox {m}$; height: $12.4\,\upmu \hbox {m}$) were extracted from single mineralised collagen fibres combining dissection, ultra-milling, pico-second pulsed laser ablation and focused ion beam (FIB) milling. First, highly mineralised tendon pieces (1.5 mm in diameter and 10.0 mm in length) were separated from tendon flexor bundles of turkey legs (*tarsometatarsus*) and fixed in aluminium sample holders using a 2-component epoxy resin adhesive (Schnellfest, UHU, Germany) after air drying the pieces for 24 h. An ultra-miller (Polycut E, Reichert-Jung, Germany) was used to polish the top surface of the tendon piece. Pico-second pulsed laser ablation (TruMicro 5250-3C, Trumpf, Germany) was used to create pre-pillars of $32.85\,\upmu \hbox {m}$ in diameter and $50.01\, \upmu \hbox {m}$ in height (laser spot size: $20\,\upmu \hbox {m}$ ($1/\hbox {e}^{2}$); pulse duration: 6 ps; pulse repetition frequency: 1 kHz, wavelength $\lambda = 515\,\hbox {nm}$; laser scanning pattern: spiral inbound anticlockwise hatch; beam overlap: 90%). Three consecutive FIB milling steps (dual FIB-SEM Quanta 3D FEG, FEI, USA) at gallium ion currents of 7.0 nA (coarse), 0.5 nA (intermediate) and 0.3 nA (polishing) were then used to cut the pre-pillars down to their final dimensions of $6.1\,\upmu \hbox {m}$ in diameter at an aspect ratio of 2.1. The reader is referred to^39^ for further details on the preparation steps, and^39^ and^110^ to show that the sample preparation procedure did not have a significant influence on the tissue properties.

### Statistical analysis

Statistical analyses were done in Python (v2.7), using the scipy^111^ and numpy^112^ packages, and R (v3.6.2)^73^. Quantile-quantile plots and Shapiro-Wilk tests were used to verify that the data were normally distributed and mean ± standard deviation were calculated. In addition, raw data are presented by means of distribution independent median, minimum and maximum values.

## Supplementary information

Supplementary sections 1-4, figures S1-S10

Supplementary video legends

Supplementary video 1a

Supplementary video 1b

Supplementary video 2a

Supplementary video 2b

Supplementary video 3
