# Supplementary material for: An experimentally informed statistical elasto-plastic mineralised collagen fibre model at the micrometre and nanometre lengthscale
Source: Sci Rep. 2021 Jul 30;11:15539. doi: 10.1038/s41598-021-93505-0 (PMC8324897; doi:10.1038/s41598-021-93505-0)
Supplement: Supplementary file 1 — Supplementary sections 1-4, figures S1-S10 [file 41598_2021_93505_MOESM1_ESM.pdf]

Supplementary material

An experimentally informed statistical elasto-plastic mineralised collagen fibre model at the micrometre and nanometre lengthscale

Alexander Groetsch, Philippe K. Zysset, Peter Varga, Alexandra Pacureanu, Francoise Peyrin, Uwe Wolfram

S1 Numerical algorithm

The analytical model (Methods sections *Two nested shear lag models for a mineralised collagen fibre* and *Elasto-plastic constitutive model for a fibril array*) was implemented in Mathematica (V11.0. Wolfram-Research, Champaign, IL, 2018). The loading protocol is given as strain versus time based on our experimental protocol [1]. Since this corresponds to a displacement-controlled compression test, the model simulates a strain-controlled test with the corresponding strain increments provided as input. The total stress at each new time step $i+1$ is calculated as the sum of n element stresses:

$$\sigma_{i+1}^{tot} = \sum_{n=1}^{N} \sigma_{i+1,n} \tag{1}$$

The same is done for the tangent operators:

$$\frac{d\sigma_{i+1}^{tot}}{d\varepsilon_{i+1}} = \sum_{n=1}^{N} \frac{d\sigma_{i+1,n}}{d\varepsilon_{i+1}} \tag{2}$$

where n denotes the n-th rheological element of a total of N elements. For every new time step, the total stress σ_{i+1}^{tot} is calculated and the internal state variables are updated based on the previous time step and a new total strain ε_{i+1}. In addition, the strain ratio distributions between the mineralised collagen fibrils and mineralised collagen fibre as well as the mineral particles and mineralised collagen fibre are calculated for each new time step $i+1$ (Methods section *Two nested shear lag models for a mineralised collagen fibre*).
The plastic strain flow for a new total strain ε_{i+1} can be computed with two trial stresses $\sigma_{i+1}^{trial,mc}$ and $\sigma_{i+1}^{trial,ef}$ based on the plastic strain in the intra- and extrafibrillar phases of the previous time step i. Since the element specific strain offset ε_{0n} is a constant (Results section *Heterogeneous deformation of fibrils due to their gradual recruitment* and Methods section *Surface roughness and fibril recruitment*) it is left out in the following descriptions:

$$\sigma_{i+1}^{trial,mc} = \epsilon(\varepsilon_i - \varepsilon_i^{p,mc} - \varepsilon_i^{p,ef}) - \chi^{mc}\varepsilon_i^{p,mc} \tag{3}$$

$$\sigma_{i+1}^{trial,ef} = \epsilon(\varepsilon_i - \varepsilon_i^{p,mc} - \varepsilon_i^{p,ef}) - \chi^{ef}\varepsilon_i^{p,ef} \tag{4}$$

Four cases need to be distinguished during the evaluation:

$$(C1) \qquad \sigma^{trial,mc} < \epsilon\varepsilon^{y,mc} \text{ and } \sigma^{trial,ef} < \epsilon\varepsilon^{y,ef} \text{fibril and matrix elastic} \tag{5}$$

$$(C2) \qquad \sigma^{trial,mc} < \epsilon\varepsilon^{y,mc} \text{ and } \sigma^{trial,ef} \geq \epsilon\varepsilon^{y,ef} \text{fibril elastic, matrix plastic} \tag{6}$$

$$(C3) \qquad \sigma^{trial,mc} \geq \epsilon\varepsilon^{y,mc} \text{ and } \sigma^{trial,ef} < \epsilon\varepsilon^{y,ef} \text{fibril plastic, matrix elastic} \tag{7}$$

$$(C4) \qquad \sigma^{trial,mc} \geq \epsilon\varepsilon^{y,mc} \text{ and } \sigma^{trial,ef} \geq \epsilon\varepsilon^{y,ef} \text{fibril and matrix plastic} \tag{8}$$

C1 *Yield criterion respected for fibrils and matrix*

No plastic flow occurs for both the mineralised collagen fibril and extrafibrillar matrix and the internal variables are updated with their value at the previous time step:

$$\varepsilon_{i+1}^{p,mc} = \varepsilon_i^{p,mc} \tag{9}$$

$$\varepsilon_{i+1}^{p,ef} = \varepsilon_i^{p,ef} \tag{10}$$

The response is elastic and the stress is given by:

$$\sigma_{i+1} = \epsilon(\varepsilon_{i+1} - \varepsilon_{i+1}^{p,mc} - \varepsilon_{i+1}^{p,ef}) = \epsilon\varepsilon_{i+1} \tag{11}$$

and the tangent operator is:

$$\frac{d\sigma_{i+1}}{d\varepsilon_{i+1}} = \epsilon \tag{12}$$

C2 *Yield criterion respected for fibrils and violated for matrix*

For the mineralised collagen fibrils, no plastic flow occurs and corresponding variables are updated with their value at the previous time step:

$$\varepsilon_{i+1}^{p,mc} = \varepsilon_i^{p,mc} \tag{13}$$

For the extrafibrillar matrix, the yield criterion is violated and we get an equation for the plastic strain $\varepsilon_{i+1}^{p,ef}$ via the implicit projection of the plastic stress on the yield criterion [2–5]:

$$f(\sigma^{p,ef}(\varepsilon_{i+1}, \varepsilon_{i+1}^{p,ef}, \varepsilon_i^{p,mc})) = 0 \tag{14}$$

The solution of this equation is:

$$\varepsilon_{i+1}^{p,ef} = \frac{\epsilon\left(\varepsilon_{i+1} - \varepsilon_i^{p,mc} - \text{sign}(\sigma_{i+1}^{trial,ef})\,\varepsilon^{y,ef}\right)}{\epsilon + \chi^{ef}} \tag{15}$$

where:

$$\text{sign}(x) = \begin{cases} -1, & x < 0 \\ 0, & x = 0 \\ 1, & x > 0 \end{cases} \tag{16}$$

The resulting stress is given by:

$$\sigma_{i+1} = \epsilon(\varepsilon_{i+1} - \varepsilon^{p,mc}_{i+1} - \varepsilon^{p,ef}_{i+1}) \tag{17}$$

and the tangent operator is then calculated as:

$$\frac{d\sigma_{i+1}}{d\varepsilon_{i+1}} = \epsilon\left(1 - \frac{\epsilon}{\epsilon + \chi^{ef}}\right) = \frac{\epsilon\chi^{ef}}{\epsilon + \chi^{ef}} \tag{18}$$

Above the ultimate strain, i.e. $|\varepsilon^{p,ef}| \geq \varepsilon^{p,ef,ult}$, the plastic slider fails and:

$$\sigma_{i+1} = 0. \tag{19}$$

C3 *Yield criterion violated for fibrils and respected for matrix*

For the extrafibrillar matrix, no plastic flow occurs and corresponding variables are updated with their value at the previous time step:

$$\varepsilon^{p,ef}_{i+1} = \varepsilon^{p,ef}_{i} \tag{20}$$

For the mineralised collagen fibrils, the yield criterion is violated and we get an equation for the plastic strain $\varepsilon^{p,mc}_{i+1}$ via the implicit projection of the plastic stress on the yield criterion (see case C2):

$$f(\sigma^{p,mc}(\varepsilon_{i+1}, \varepsilon^{p,ef}_{i+1}, \varepsilon^{p,mc}_{i+1})) = 0 \tag{21}$$

The solution of this equation is:

$$\varepsilon^{p,mc}_{i+1} = \frac{\epsilon\left(\varepsilon_{i+1} - \varepsilon^{p,ef}_{i} - \text{sign}(\sigma^{trial,mc}_{i+1})\ \varepsilon^{y,mc}\right)}{\epsilon + \chi^{mc}} \tag{22}$$

The resulting stress is given by:

$$\sigma_{i+1} = \epsilon(\varepsilon_{i+1} - \varepsilon^{p,mc}_{i+1} - \varepsilon^{p,ef}_{i+1}) \tag{23}$$

and the tangent operator is:

$$\frac{d\sigma_{i+1}}{d\varepsilon_{i+1}} = \epsilon\left(1 - \frac{\epsilon}{\epsilon + \chi^{mc}}\right) = \frac{\epsilon\chi^{mc}}{\epsilon + \chi^{mc}} \tag{24}$$

Above the ultimate strain, i.e. $|\varepsilon^{p,mc}| \geq \varepsilon^{p,mc,ult}$, the plastic slider fails and:

$$\sigma_{i+1} = 0. \tag{25}$$

C4 *Yield criteria violated for fibrils and matrix*

The yield criterion is violated for both the mineralised collagen fibrils and extrafibrillar matrix. We, thus, consider a coupled plastic flow and equations for the plastic strains at the time step $i+1$. In this case, we need to perform simultaneous implicit projections of the plastic stresses on the yield criteria for the intra- and extrafibrillar phases to get equations for the updated plastic strains $\varepsilon^{p,ef}_{i+1}$ and $\varepsilon^{p,mc}_{i+1}$:

$$f(\sigma^{p,ef}(\varepsilon_{i+1}, \varepsilon^{p,ef}_{i+1}, \varepsilon^{p,mc}_{i+1})) = 0 \tag{26}$$

$$f(\sigma^{p,mc}(\varepsilon_{i+1}, \varepsilon^{p,ef}_{i+1}, \varepsilon^{p,mc}_{i+1})) = 0 \tag{27}$$

The solutions of this equation are:

$$\varepsilon^{p,ef}_{i+1} = \frac{\epsilon\left(\varepsilon_{i+1}\,\chi^{mc} - \varepsilon^{y,ef}(\epsilon + \chi^{mc})\,\mathrm{sign}(\sigma^{trial,ef}_{i+1}) + \epsilon\varepsilon^{y,mc}\,\mathrm{sign}(\sigma^{trial,mc}_{i+1})\right)}{\chi^{ef}\chi^{mc} + \epsilon(\chi^{ef} + \chi^{mc})} \tag{28}$$

$$\varepsilon^{p,mc}_{i+1} = \frac{\epsilon\left(\varepsilon_{i+1}\,\chi^{ef} + \epsilon\varepsilon^{y,ef}\,\mathrm{sign}(\sigma^{trial,ef}_{i+1}) - \varepsilon^{y,mc}(\epsilon + \chi^{ef})\,\mathrm{sign}(\sigma^{trial,mc}_{i+1})\right)}{\chi^{ef}\chi^{mc} + \epsilon(\chi^{ef} + \chi^{mc})} \tag{29}$$

The resulting stress is given by:

$$\sigma_{i+1} = \epsilon(\varepsilon_{i+1} - \varepsilon^{p,mc}_{i+1} - \varepsilon^{p,ef}_{i+1}) \tag{30}$$

and the tangent operator is:

$$\frac{d\sigma_{i+1}}{d\varepsilon_{i+1}} = \epsilon\left(1 - \frac{\epsilon\chi^{ef}}{\chi^{ef}\chi^{mc} + \epsilon(\chi^{ef} + \chi^{mc})} - \frac{\epsilon\chi^{mc}}{\chi^{ef}\chi^{mc} + \epsilon(\chi^{ef} + \chi^{mc})}\right) \tag{31}$$

$$= \frac{\epsilon\chi^{ef}\chi^{mc}}{\chi^{ef}\chi^{mc} + \epsilon(\chi^{ef} + \chi^{mc})} \tag{32}$$

Above ultimate strains $|\varepsilon^{p,ef}| \geq \varepsilon^{p,ef,ult}$ and $|\varepsilon^{p,mc}| \geq \varepsilon^{p,mc,ult}$, the plastic sliders fail and:

$$\sigma_{i+1} = 0. \tag{33}$$

S2 Simulation steps for different loading stages

S2.1 Mineralised collagen fibril recruitment and failure

In the following, we provide the model outcome at different loading steps, primarily to illustrate the differences between the models with and without non-linear recruitment of mineralised collagen fibrils (Figures S1 to S6 with an overview in Table S1). The full loading cycles can be found in the Supplementary Videos 1 and 2 which also includes an overlay with experimental curves (Videos 1b, 2b). Figures S1 to S6 cover loading steps of the simulated fibre behaviour at the numerical yield point, at full recruitment and when mineralised collagen fibrils fail. Grey lines denote experimental values [1]. Images (a) to (d) always denote the following: (a) experimental loading protocol used also for the simulations; (b) simulated stress-strain curve for the mineralised collagen fibre up to a mean maximum strain of 12%; (c) statistical distribution for the strain ratios between mineralised collagen fibrils and the apparent mineralised collagen fibre; (d) strain ratio distributions between the mineral particles and mineralised collagen fibre. Each page shows the same loading step in the recruitment and non-recruitment model outcome. For the recruitment model, we saw that at the numerical yield strain of 0.04, there are still around 35 unrecruited mineralised collagen fibrils (Figure S1). No recruitment takes place in the non-recruitment model and no counts at 0 in the elastic region are visible (Figure S2). After full contact has been established between the flat punch and the micropillar, counts drop to zero for the recruitment model (Figures S3c,d). At the same loading step, the first mineralised collagen fibrils start to fail in the non-recruitment model. (Figures S4c,d). With an increasing compressive stress, an increasing number of mineralised collagen fibrils fail. At an apparent fibre strain of around 0.09, about four times as many mineralised collagen fibrils have already failed when we disregard the gradual fibril recruitment (Figures S6c,d) compared to the case when we consider it (Figures S5c,d).

Table S1: Six figures outline the details on the mineralised collagen fibril recruitment and failure including loading steps at the numerical yield point, at full recruitment and when fibrils fail.

	numerical yield point	full recruitment	fibrils fail
with fibril recruitment	Figure S1	Figure S3	Figure S5
without fibril recruitment	Figure S2		Figures S2, S6

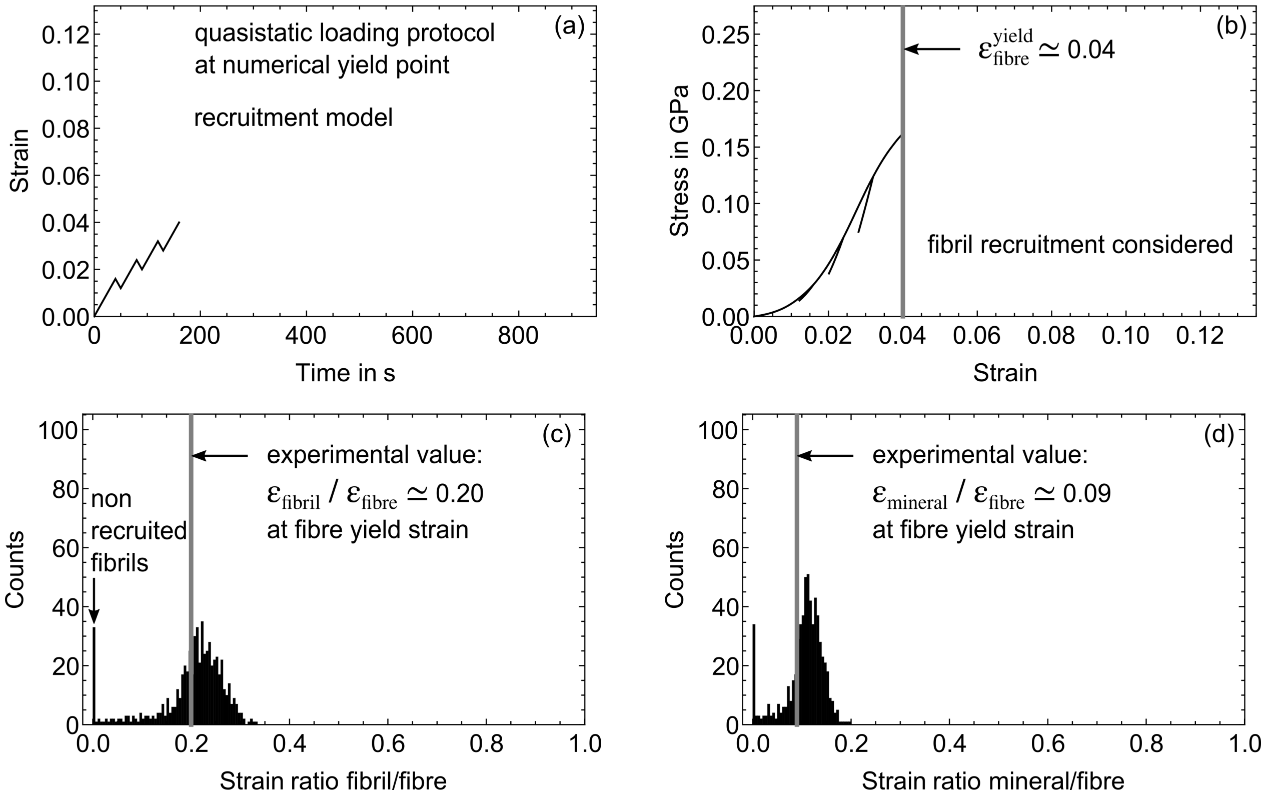

Figure S1: Model with mineralised collagen fibril recruitment at the numerical yield point showing unrecruited mineralised collagen fibrils and an agreement with experiments [1]. Still not all fibrils carry load.

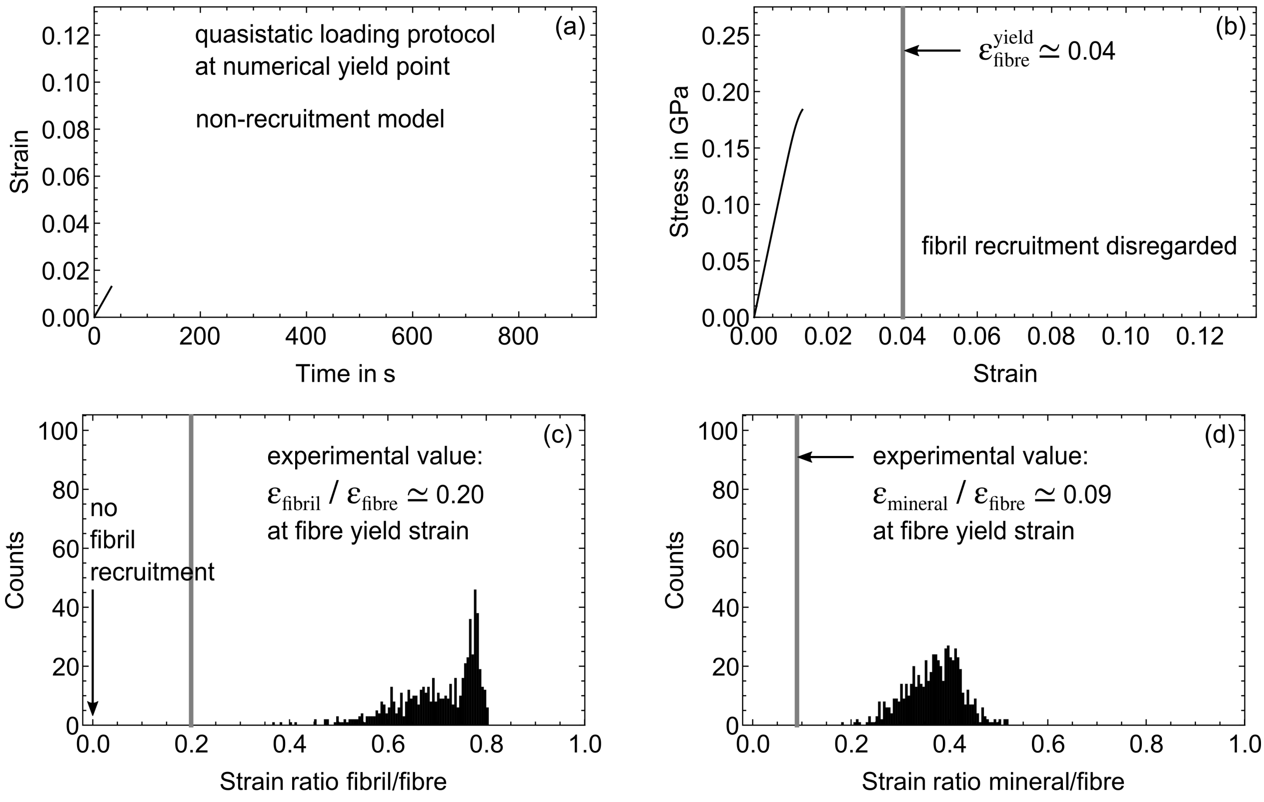

Figure S2: Model without mineralised collagen fibril recruitment at the numerical yield point showing no recruitment and a disagreement with experiments [1]. All fibrils carry load.

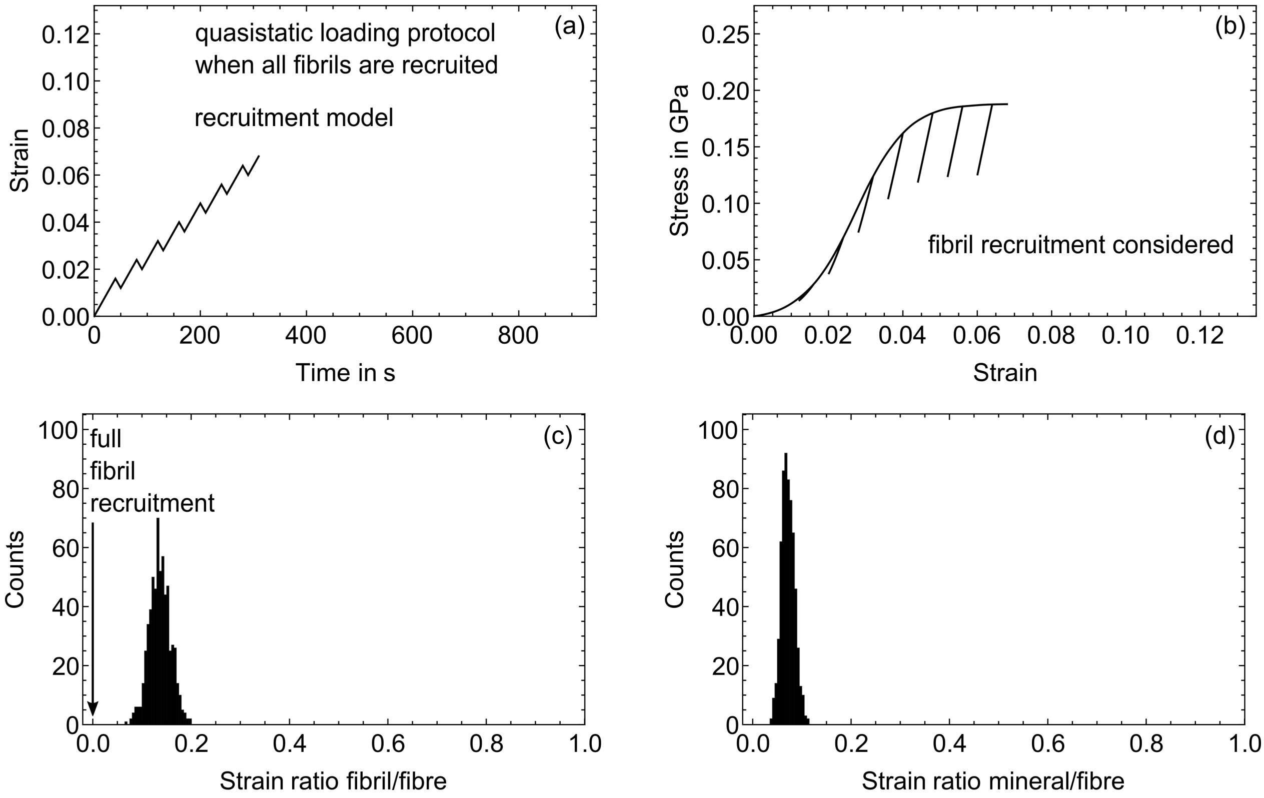

Figure S3: Model with mineralised collagen fibril recruitment at a point of full recruitment in the plastic region of the compressed mineralised collagen fibre. All fibrils carry load.

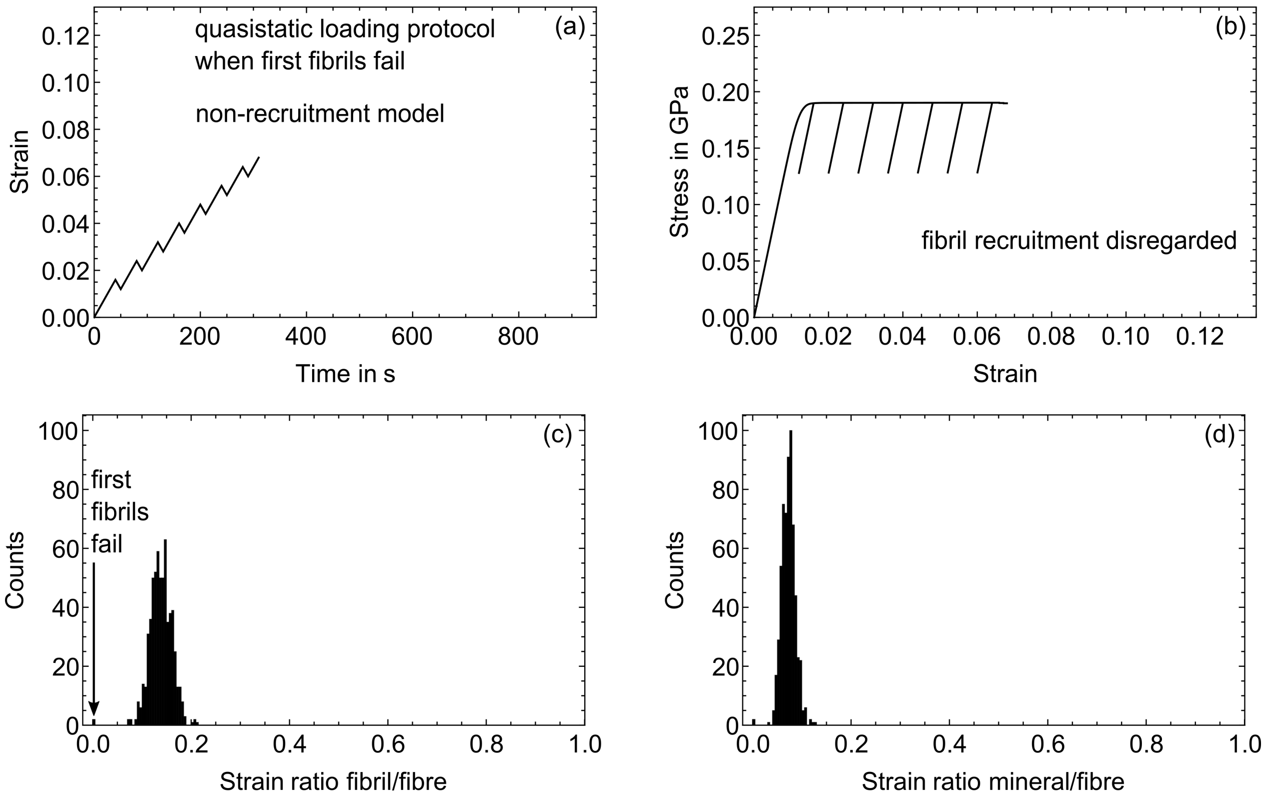

Figure S4: Model without mineralised collagen fibril recruitment at a point when the first fibrils fail at the same loading step when in the recruitment model (Figures S3c,d) all fibrils have only just been recruited.

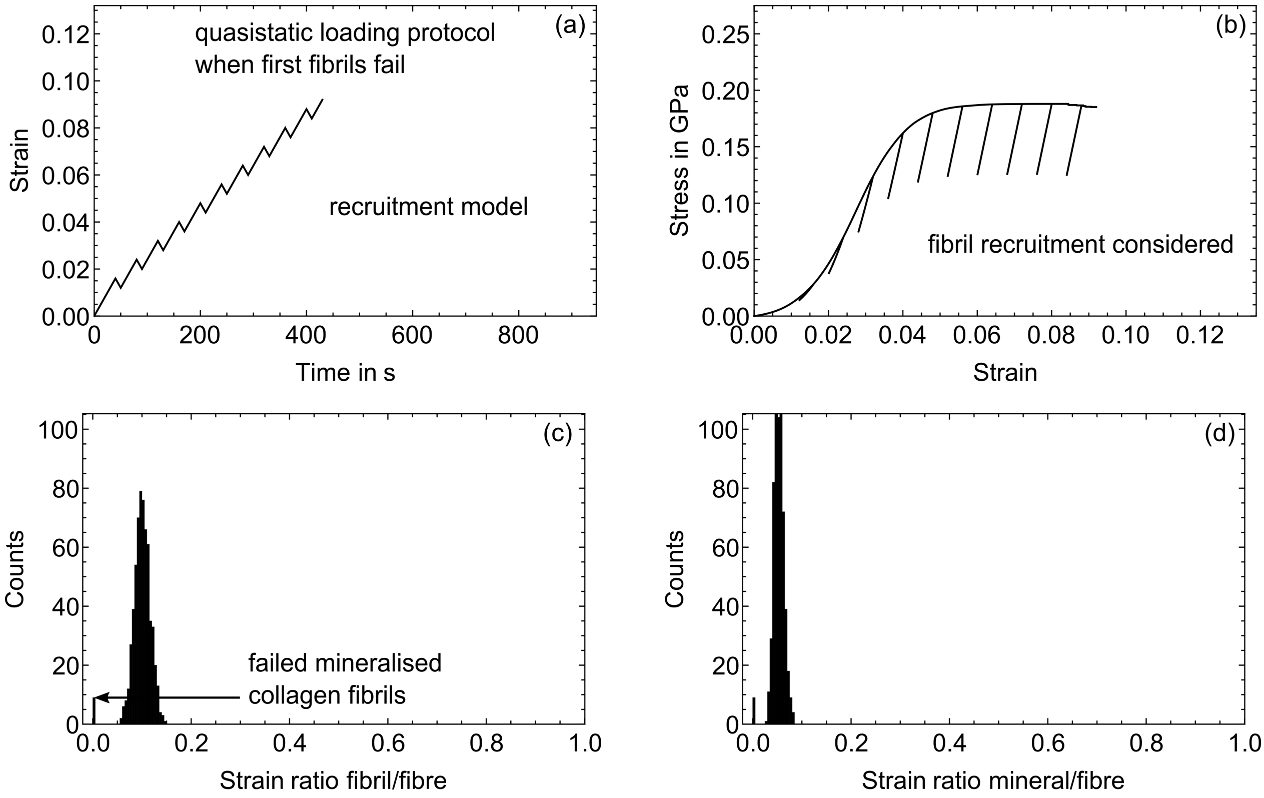

Figure S5: Model with mineralised collagen fibril recruitment at a point in the plastic region of the mineralised collagen fibre when fibrils start to fail.

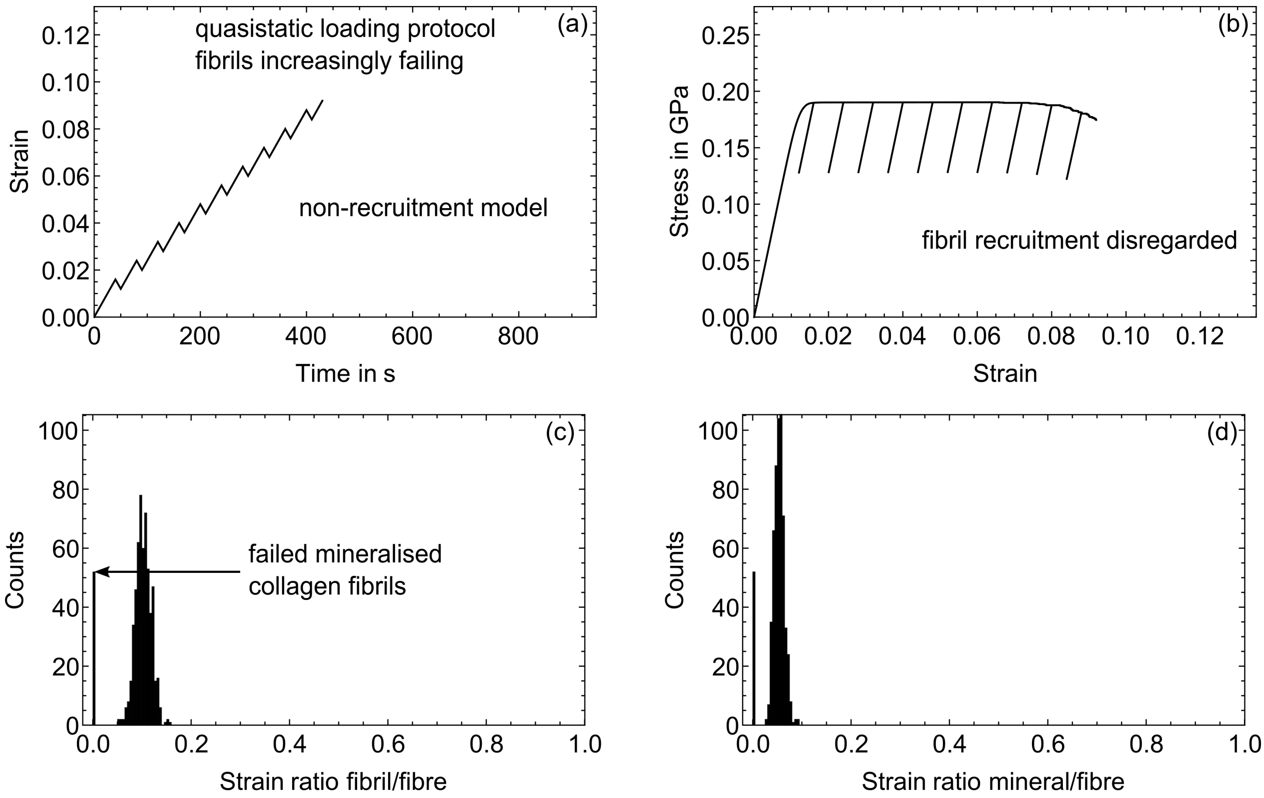

Figure S6: Model without mineralised collagen fibril recruitment at a point when an increasing number of fibrils fail (same loading step as in Figure S5).

S3 Synchrotron radiation phase-contrast nanoCT (SRnCT)

In the following, we provide details on the SRnCT theory, measurements and analysis. During an SRnCT experiment, the interaction of the X-ray beam with the sample is modelled by the complex refractive index $n(x, y, z) = 1 - \delta_{dec} + i\beta$ where β denotes the absorption index and δ_{dec} the refractive index decrement. During the scan, the real part of the refractive index n is integrated along the propagation direction z yielding a modulation of the beam amplitude $A(x, y) = \frac{2\,\pi}{\lambda} \int \beta(x, y, z)\, dz$ and a phase shift of the phase $\varphi(x, y) = -\frac{2\,\pi}{\lambda} \int \delta_{dec}(x, y, z)\, dz$. The phase contribution is encoded in the recorded intensity (phase contrast projections). While β is proportional to the linear attenuation coefficient μ via $\mu = \frac{4\pi\,\beta}{\lambda}$, $\delta_{dec} = \frac{r_c\,\lambda}{2\pi\,V} \sum_j (Z_j + f_j)$ contains the information that enables a high sensitivity for light organic elements by summing over all atoms contained in the representative volume V (Z_i: atomic number, f_j: real part of the dispersion correction for the specific X-ray wavelength; r_c = 2.8 fm: classical radius of the electron). This high sensitivity allows it to detect structural differences at the nanoscale which was necessary for informing our micro- and nanomechanical model. If we use an X-ray energy that lies above the absorption edges of the sample's constitutive elements, we can neglect any dispersion correction and δ_{dec} is directly proportional to the sample's electron density ρ. By using hard X-rays, we can, thus, neglect propagation and dynamical effects inside the sample. In the case of mostly light elements, such as in bone, a ratio of the atomic number over the atomic mass Z/M is observed of being very close to $1/2$ with Z as the number of electrons in the atomic group of atomic mass M. With the Guinier approximation [6], we get $\delta_{dec} = 2.72 \cdot 10^{-6}\, \frac{Z}{M}\, \rho\, \lambda^2$ and $\rho = -\frac{1}{2\pi}\, \frac{\omega}{1.3\,\lambda} \cdot 10^{-2}$.

During the SRnCT measurements, four distances between the sample and the focal spot at various projection angles were recorded. First, for each angle, the phase contrast images were aligned using a mutual information-based code [7]. Second, the phase retrieval process was applied to all four images per projection angle followed by an iterative non-linear minimisation step. We used the multi Paganin approach [8] which is a linear filtering algorithm followed by an iterative non-linear refinement step. The phase retrieval parameters are optimised interactively while working on one rotation angle. Succeeding angles can be processed via a batch processing in Python (v2.7). These phase maps are further processed with a tomographic reconstruction algorithm by means of a filtered backprojection [9] using the PyHST software developed at the ESRF. One sample showed extremely large misalignments (>800 pixels), either due to motor drift of unknown origin or an incorrectly inserted Huber pin sample holder into the rotation stage, with a large part of the sample but not the actual micropillar exiting the field of view, thus requiring manual registration. For this sample, phase retrieval for pure phase objects (CTF without attenuation term) without iterative refinement was used. This was motivated by the low attenuation in the thin micropillars. Iterative refinement led to slightly higher resolution at the cost of increased noise and artefacts due to the low signal to noise (SNR) in the recorded images. A δ/β ratio of 203, corresponding to cortical bone was used to correct the DC component. Supplementary Video 3 shows the cross-sectional slicing through an exemplary sample illustrating (i) the surface roughness by the gradual appearance of the FIB-milled micropillar (post-test), (ii) the laser ablated pre-pillar and the (iii) volume below.

S3.1 Tissue density of a mineralised collagen fibre

Figure S7 informs about the tissue density calculation via probability density functions of grey value regions (Results section *Tissue mineral density within a fibre* and Methods section *Tissue mineral density within a fibre*). Figure S8 shows the selection of the volume of interest (VOI) to calculate the global offset value. Regions A, B, C within the SRnCT image denote areas where the X-ray beam did not directly interact with the sample (background signal). A gradient along the z-axis was detectable. The region at the base next to the micropillar was used to offset correct the tissue density values.

S3.2 Local 3D mineralised collagen fibril orientation

The local 3D orientation of the mineralised collagen fibrils were quantified by means of an auto-correlation based measure [10] (Results section *Parallel fibril arrangement within a fibre* and Methods section *Local 3D fibril orientation*). It confirmed the parallel arrangement of the fibrils, which justified the use of a parallel arrangement of single model elements in our simulations. The plastic deformation of the compressed micropillars at the top part resulted in a deviation from the longitudinal arrangement (Figure S9). Post-test SEM images showed us that the top surface was planar with a certain degree of roughness. Since we detected a deformation localisation for the tested micropillars [1], the compression has likely reduced the roughness by plastic deformation. Figure S9 shows the streamline and vectorial representations that visualise the direction of the fibrils and the degree of anisotropy of the auto-correlation measure used as the fidelity measure of the evaluated orientations following Varga et al. [10].

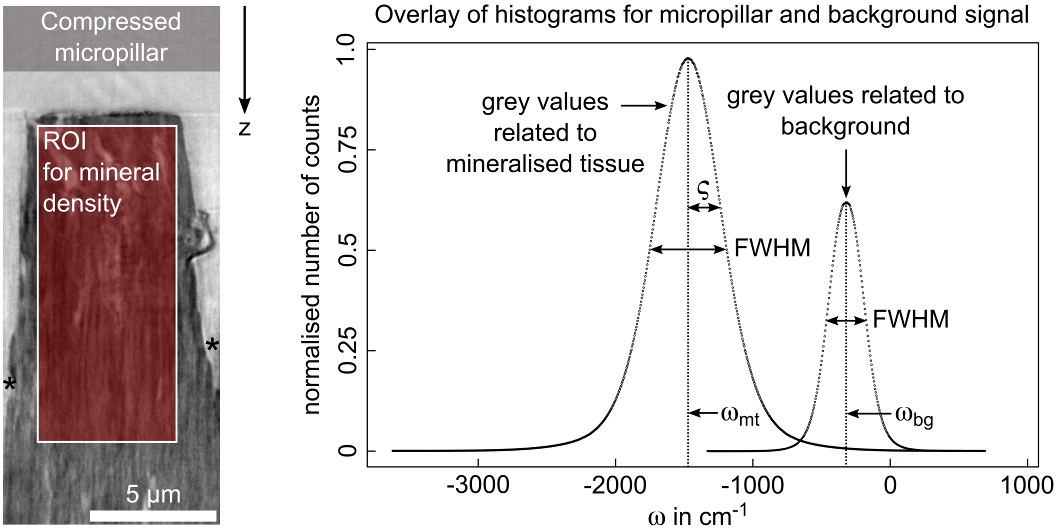

Figure S7: Left: Sagittal view of a reconstructed SRnCT of a compressed micropillar. The micropillar tissue density was determined in regions of interest (ROIs) within the micropillar. Asterisks denote the background signal (region C in Figure S8). Right: Overlay of both histograms, for mineralised tissue and the background. Peaks were identified by a Gaussian fit and initial peak position values via a custom written code in R (v3.6.2). ς denotes the standard deviation of the Gaussian fit, ω_{mt} the mean value for the mineralised tissue, ω_{bg} the mean value for the background. ω_{bg} was used as a global offset for the reconstructed values. The FWHM of the Gaussian fit were used to calculate the standard deviation of the ω - values.

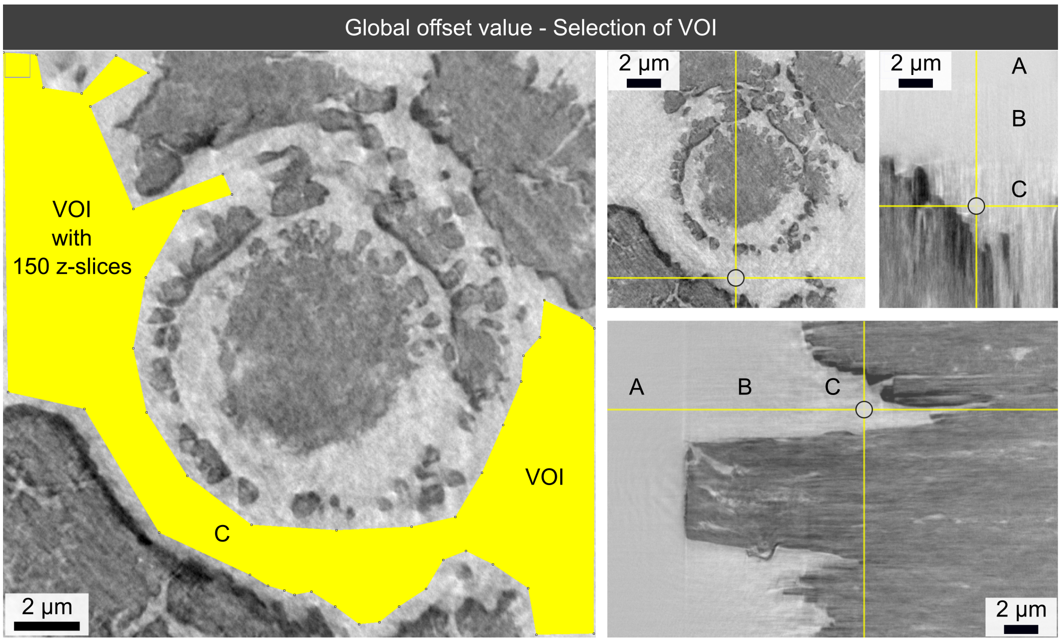

Figure S8: The ω - values in different non-mineralised regions showed a decreasing gradient towards the base of the micropillar (regions A, B, C). The yellow region (left) indicates the volume of interest (VOI) (region C) with a depth of 150 slices (z-direction). This depth can be seen in the bottom right image. The VOI was used to calculate the probability density function of the background signal and the offset value ω_{bg} (Figure S7). The circular regions (crossing yellow lines; right) denote the same location in different views.

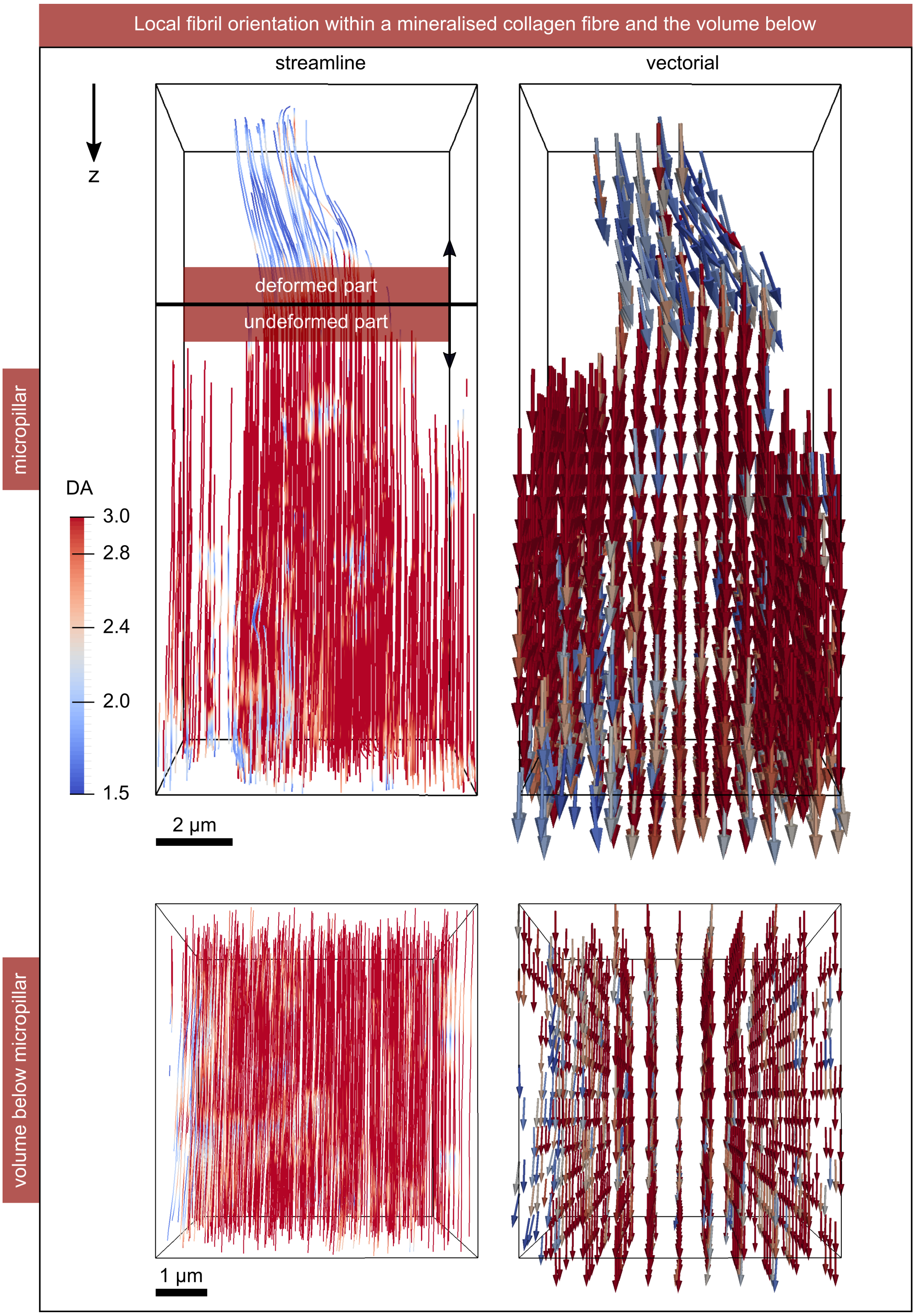

Figure S9: 3D mineralised collagen fibril orientation of a compressed micropillar and the volume below. Streamline and vectorial representations show the off-axis angles of the orientation vectors. The misalignment in the micropillar top part (deformed part) is related to strain localisation during compression. For the alignment analysis, the undeformed micropillar part and the volume below were used. DA = degree of anisotropy. See Methods section *Local 3D fibril orientation* for a detailed description of the analysis. Visualisations done in Paraview [11].

S4 Influence of using a skewed statistical distribution

In Methods section *Statistical material properties*, we motivate the use of a normal distribution with a standard deviation of 15% to represent the microscale heterogeneity in our model. To confirm the suitability of this choice, we studied the influence that a skewed distribution has on the simulated fibre behaviour. Two skewed distributions with the same median were chosen, one skewed towards smaller values and one towards larger values (Figure S10). The outcome was compared to results when using a normal distribution (Results section *Simulated fibre with fibril recruitment*). Mean values (expectancy values) and standard deviations were the same for all three simulation runs. The variation analysis shows that the use of a skewed distribution does not result in significantly different strain ratios or stiffness and only minor changes in strength. We, thus, conclude that skewed distributions do not present an advantageous set of properties for the material system. Compared to the 95% overall agreement between model and experiments when using a normal distribution (Table 1, main text), we observe an agreement of 93% when skewed towards smaller values and 90% when skewed towards larger values. Based on all mechanical data including our own experiments, and the used parameters in our model, we can assume a normal distribution.

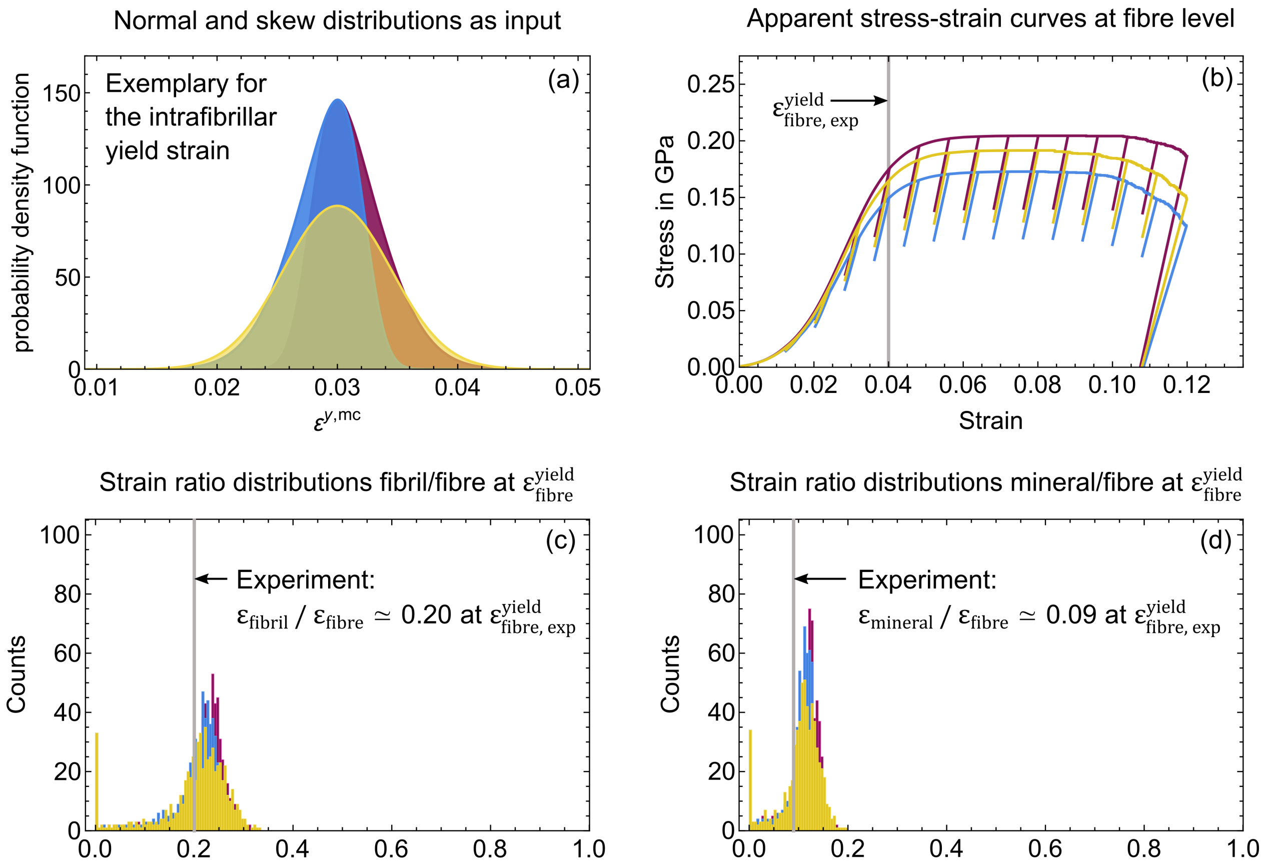

Figure S10: Influence of different statistical distributions on the simulation outcome. (a) Normal and skewed distributions, here exemplary for the intrafibrillar yield strain $\varepsilon^{y,mc}$. The same type of statistical distribution was used for all mechanical properties for each simulation run. The colour coding applies to all subplots. (b) Influence on the apparent behaviour (stress-strain data) at mineralised collagen fibre level. (c) Influence on the fibril-to-fibre strain ratio distributions at the experimental yield point. (d) Influence on the mineral-to-fibre strain ratio distributions at the experimental yield point.

References

[1] A. Groetsch, A. Gourrier, J. Schwiedrzik, M. Sztucki, R. J. Beck, J. D. Shephard, J. Michler, P. K. Zysset, and U. Wolfram. Compressive behaviour of uniaxially aligned individual mineralised collagen fibres at the micro- and nanoscale. *Acta Biomaterialia*, 89:313 – 329, 2019.

[2] P. K. Zysset. *A constitutive law for trabecular bone*. PhD thesis, École Polytechnique Fédérale de Lausanne, 1994.

[3] P. K. Zysset and A. Curnier. An implicit projection algorithm for simultaneous flow of plasticity and damage in standard generalized materials. *Int J Num Meth*, 39:3065 – 3082, 1996.

[4] J. C. Simo and T. J. R. Hughes. *Computational Inelasticity*. Springer, 2000.

[5] J. J. Schwiedrzik, R. Raghavan, A. Bürki, V. LeNader, U. Wolfram, J. Michler, and P. K. Zysset. In situ micropillar compression reveals superior strength and ductility but an absence of damage in lamellar bone. *Nature Materials*, 13(7):740 – 747, 2014.

[6] A. Guinier. *X-Ray Diffraction - In Crystals, Imperfect Crystals, and Amorphous Bodies*. W. H. Freeman and Company, San Francisco and London, 1963.

[7] L. Weber, M. Langer, S. Tavella, A. Ruggiu, and F. Peyrin. Quantitative evaluation of regularized phase retrieval algorithms on bone scaffolds seeded with bone cells. *Physics in Medicine and Biology*, 61(9):N215–231, 2016.

[8] D. Paganin, S. C. Mayo, T. E. Gureyev, P. R. Miller, and S. W. Wilkins. Simultaneous phase and amplitude extraction from a single defocused image of a homogeneous object. *Journal of Microscopy*, 206(1):33–40, 2002.

[9] P. Varga, B. Hesse, M. Langer, S. Schrof, N. Männicke, H. Suhonen, A. Pacureanu, D. Pahr, F. Perin, and K. Raum. Synchrotron x-ray phase nano-tomography-based analysis of the lacunar–canalicular network morphology and its relation to the strains experienced by osteocytes in situ as predicted by case-specific finite element analysis. *Biomechanics and Modeling in Mechanobiology*, 14(2):267–282, 2015.

[10] P. Varga, A. Pacureanu, M. Langer, H. Suhonen, B. Hesse, Q. Grimal, P. Cloetens, K. Raum, and F. Peyrin. Investigation of the 3D orientation of mineralized collagen fibrils in human lamellar bone using synchrotron x-ray phase nano-tomography. *Acta Biomat*, 9:8118–27, 2013.

[11] J. Ahrens, V. Geveci, and C. Law. *ParaView: An End-User Tool for Large Data Visualization, Visualization Handbook*. Number ISBN-13: 978-0123875822. Elsevier, 2005.
