# Supplementary material for: An experimentally informed statistical elasto-plastic mineralised collagen fibre model at the micrometre and nanometre lengthscale
Source: Sci Rep. 2021 Jul 30;11:15539. doi: 10.1038/s41598-021-93505-0 (PMC8324897; doi:10.1038/s41598-021-93505-0)
Supplement: Supplementary file 2 — Supplementary video legends [file 41598_2021_93505_MOESM2_ESM.pdf]

Video descriptions

Five videos in total
Main video for the comparison between model and experiment: 2b

In the videos 1a, 1b, 2a and 2b, we always provide the loading protocol (top left), the apparent fibre behaviour (top right), the statistical distribution for the load transfer between fibrils and fibre (bottom left) and the statistical distribution for the load transfer between mineral nanocrystals and the fibre (bottom right).

Groetsch-et-al_2021_Video1a_Model_withoutFibrilRecruitment.mp4
In this video we show the simulated behaviour of a mineralised collagen fibre as a result of our statistical constitutive model. Please see the description of Video 1b for a comparison with experiments and Videos 2a and 2b for the versions where we consider a non-linear recruitment mechanism of mineralised collagen fibrils during compression. Further details are provided in the corresponding video descriptions.

Groetsch-et-al_2021_Video1b_Model-vs-Experiment_withoutFibrilRecruitment.mp4
The video compares the simulated behaviour of a mineralised collagen fibre from our model (without fibril recruitment) with in situ micromechanical and synchrotron experiments. Please see the description of Videos 2a and 2b for further information.

Groetsch-et-al_2021_Video2a_Model_withFibrilRecruitment.mp4
In this video we show the simulated behaviour of a mineralised collagen fibre as a result of our statistical constitutive model when we consider a non-linear recruitment of mineralised collagen fibrils. Please see the description of 2b for a comparison with experiments and further information

Groetsch-et-al_2021_Video2b_Model-vs-Experiment_withFibrilRecruitment.mp4
The video shows the high agreement between the simulated behaviour of a mineralised collagen fibre when we compare the results from our statistical constitutive model with our in situ micromechanical and synchrotron experiments. Here, we consider that mineralised collagen fibrils are being gradually recruited in a non-linear fashion when we compress the fibre. We identified this fibril recruitment mechanism by means of synchrotron radiation X-ray phase-contrast nanometre computed tomography with a voxel size of 20 nm. Video 3 shows a cross-section slicing through a fibre micropillar and the volume below. Since model and experiment address the same hierarchical levels of bone, and the model outputs a statistical distribution of strain values, a direct comparison with in situ micromechanical and synchrotron X-ray scattering experiments (SAXS, XRD) is possible. It allows us to explore and directly compare the micro- and nanomechanical behaviour of bone's fundamental building block as seen in experiments and in simulations.

Groetsch-et-al_2021_Video3_SRnCT_SynchrotronNanoscaleImaging.mp4
In this video, we show the axial slicing through the micropillar extracted from a single mineralised collagen fibre. The slices were reconstructed from synchrotron radiation X-ray phase-contrast nanometre computed tomography where we scanned the samples with a voxel size of 20 nm. This resolution allowed us to identify a mechanisms that led to a gradual and non-linear recruitment of mineralised collagen fibrils upon compression. We implemented this mechanism in our model via a sigmoid function and achieved a very high agreement with experiments.
